# cAMP- and cGMP-elevating agents inhibit GPIbα-mediated aggregation but not GPIbα-stimulated Syk activation in human platelets

**DOI:** 10.1186/s12964-019-0428-1

**Published:** 2019-09-13

**Authors:** Stephanie Makhoul, Katharina Trabold, Stepan Gambaryan, Stefan Tenzer, Daniele Pillitteri, Ulrich Walter, Kerstin Jurk

**Affiliations:** 1 Center for Thrombosis and Hemostasis (CTH), University Medical Center Mainz of the Johannes Gutenberg University Mainz, Mainz, Germany; 2 Sechenov Institute of Evolutionary Physiology and Biochemistry, Russian Academy of Sciences, St. Petersburg, Russia; 3 Core Facility for Mass Spectrometry, Institute for Immunology, University Medical Center Mainz, Mainz, Germany; 4 DKD HELIOS Klinik Wiesbaden GmbH, Wiesbaden, Germany

**Keywords:** cAMP-dependent protein kinase, cGMP-dependent protein kinase, Glycoprotein receptor GPIb-IX, Platelet activation, Syk kinase

## Abstract

**Background:**

The glycoprotein (GP) Ib-IX-V complex is a unique platelet plasma membrane receptor, which is essential for platelet adhesion and thrombus formation. GPIbα, part of the GPIb-IX-V complex, has several physiological ligands such as von Willebrand factor (vWF), thrombospondin and distinct coagulation factors, which trigger platelet activation. Despite having an important role, intracellular GPIb-IX-V signaling and its regulation by other pathways are not well defined. Our aim was to establish the intracellular signaling response of selective GPIbα activation in human platelets, in particular the role of the tyrosine kinase Syk and its regulation by cAMP/PKA and cGMP/PKG pathways, respectively. We addressed this using echicetin beads (EB), which selectively bind to GPIbα and induce platelet aggregation.

**Methods:**

Purified echicetin from snake *Echis carinatus* venom was validated by mass spectrometry. Washed human platelets were incubated with EB, in the presence or absence of echicetin monomers (EM), Src family kinase (SFK) inhibitors, Syk inhibitors and the cAMP- and cGMP-elevating agents iloprost and riociguat, respectively. Platelet aggregation was analyzed by light transmission aggregometry, protein phosphorylation by immunoblotting. Intracellular messengers inositolmonophosphate (InsP1) and Ca^2+^_i_ were measured by ELISA and Fluo-3 AM/FACS, respectively.

**Results:**

EB-induced platelet aggregation was dependent on integrin α_IIb_β_3_ and secondary mediators ADP and TxA_2_, and was antagonized by EM. EB stimulated Syk tyrosine phosphorylation at Y352, which was SFK-dependent and Syk-independent, whereas Y525/526 phosphorylation was SFK-dependent and partially Syk-dependent. Furthermore, phosphorylation of both Syk Y352 and Y525/526 was completely integrin α_IIb_β_3_-independent but, in the case of Y525/526, was partially ADP/TxA_2_-dependent. Syk activation, observed as Y352/ Y525/Y526 phosphorylation, led to the phosphorylation of direct substrates (LAT Y191, PLCγ2 Y759) and additional targets (Akt S473). PKA/PKG pathways inhibited EB-induced platelet aggregation and Akt phosphorylation but, surprisingly, enhanced Syk and LAT/PLCγ2 tyrosine phosphorylation. A similar PKA/PKG effect was confirmed with convulxin−/GPVI-stimulated platelets. EB-induced InsP1 accumulation/InsP3 production and Ca^2+^-release were Syk-dependent, but only partially inhibited by PKA/PKG pathways.

**Conclusion:**

EB and EM are specific agonists and antagonists, respectively, of GPIbα-mediated Syk activation leading to platelet aggregation. The cAMP/PKA and cGMP/PKG pathways do not inhibit but enhance GPIbα−/GPVI-initiated, SFK-dependent Syk activation, but strongly inhibit further downstream responses including aggregation. These data establish an important intracellular regulatory network induced by GPIbα.

**Graphical abstract:**

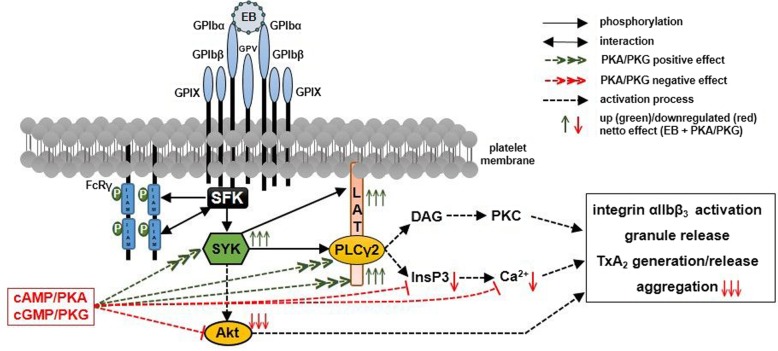

**Electronic supplementary material:**

The online version of this article (10.1186/s12964-019-0428-1) contains supplementary material, which is available to authorized users.

## Plain English summary

As the smallest circulating blood cells, platelets are activated at sites of vascular injury resulting in adhesion, aggregation, and blood coagulation, thereby preventing major blood loss. Pathologically, platelet hypofunction and hyperfunction can result in life-threatening bleeding or thromboinflammatory disorders, respectively. Platelet activation responses with subsequent thrombus formation are tightly controlled by multiple factors, which promote or inhibit platelet activation via membrane receptors and their intracellular effector systems. An essential platelet receptor is the glycoprotein (GP) Ib-IX-V complex, and its subunit GPIbα ligates predominantly von Willebrand factor but also other adhesion proteins and distinct coagulation factors. This enables platelet recruitment to the vessel wall, aggregation and coagulation. Despite this important role, intracellular effects of GPIb-IX-V in platelets and their interaction with other signaling pathways are not well defined. Recently, we characterized the snake venom protein echicetin immobilized on polystyrene beads as a specific GPIb activator. With this tool, we now show that GPIbα causes the activation of the tyrosine kinase Syk in a Src family kinase dependent manner, which results in the generation of further intracellular messengers and ultimately platelet aggregation. Surprisingly, activation of the platelet inhibitory cAMP/PKA and cGMP/PKG pathways enhanced initial Syk phosphorylation/activation, but strongly inhibited GPIbα-induced platelet aggregation, which is distal to Syk activation. These studies establish a new intracellular regulatory network triggered by the activation of GPIbα in human platelets.

## Background

Circulating platelets are essential for both physiological and pathological hemostasis and have important roles in inflammatory diseases and cancer [1–3]. Platelet activating, inhibitory and modulating factors fine tune platelet adhesion to the vessel wall. Physiologically, this fine-tuning prevents excessive bleeding due to vascular injuries, and also prevents or limits pathological thrombus formation / vessel occlusion at sites of injured blood vessels.

Two major groups of platelet activators such as soluble agonists [thrombin, ADP, thromboxane A_2_ (TxA_2_)] and adhesion molecules [e.g. von Willebrand factor (vWF), collagen, fibrin, podoplanin] bind to and stimulate specific G-protein-coupled receptors (GPCRs), or cell membrane-spanning adhesion receptors, respectively. These receptors stimulate intracellular signaling pathways and various platelet responses leading to integrin activation (e.g. integrin α_IIb_β_3_), granule secretion, exposure of anionic phospholipids [4–6] and subsequently firm adhesion, aggregation, thrombin generation and thrombus formation. Conversely, elevation of platelet cAMP or cGMP by endothelial-derived prostacyclin (PGI_2_) or nitric oxide (NO) inhibits via cAMP-dependent (PKA) and/or cGMP-dependent (PKG) protein kinases, respectively, many of these platelet activation responses at several sites of the activation pathways [7–9]. Activation of Src-family kinases (SFK) and subsequent protein tyrosine phosphorylation, including membrane proteins containing the “immunoreceptor tyrosine-based activation motif (ITAM)”, initiates receptor-mediated platelet activation via the GPVI/Fcγ chain [10–13], integrin α_IIb_β_3_/FcγRIIA, CLEC-2, and GPIb-V-IX [5, 14, 15].

ITAM-mediated Syk activation, discovered in immune cells [16, 17], is now established in many mammalian cells including platelets [5, 11, 18]. For human platelets, the presence of two ITAM proteins have been described, Fc receptor γ-chain (FcRγ; gene: FCER1G) and a low affinity IgG receptor FcγRIIa (gene: FCGR2A) [19, 20]. In murine platelets only the FcRγ has been described [21, 22]. Studies with platelets from FcRγ-deficient mice established that this protein is essential for GPVI expression and function [12]. Cytosolic Syk is activated by two distinct overlapping mechanisms designated as ITAM-dependent or Y-phosphorylation-dependent [18, 23–25]. The Syk Y-phospho-sites Y348/ Y352 and Y525/ Y526 belong to 2 pairs within the interdomain linker and kinase domain, respectively. Syk activation is initiated when these Y-sites are phosphorylated by SFKs or when dually Y-phosphorylated ITAM-containing membrane proteins recruit the two Syk-SH2 domains followed by Syk autophosphorylation [23, 24]. Usually, Syk activation is analyzed by Syk tyrosine phosphorylation (pY352 and pY525/526) and as tyrosine phosphorylation of its substrates (LAT pY191, PLCγ2 pY759). However, there are many additional proteins known to be directly phosphorylated by Syk [26–28]. Over the last years, hundreds of Syk targets have been identified in proteomic/phosphoproteomic studies and used to generate distinct Syk networks in cancer cells [28, 29], and many of these Syk targets are present in human platelets.

vWF and its major receptor, the glycoprotein Ib-V-IX (GPIb-IX-V) complex, are essential for platelet adhesion and initial thrombus formation at sites of vascular injury under arterial and venous shear conditions [20, 30, 31]. The GPIb-IX-V complex consists of 4 transmembrane proteins (GPIbα, GPIbß, GPIX, GPV; stoichiometry of 2:2:2:1). GPIbα is of special importance since it binds most of the extracellular GPIb-IX-V complex ligands (e.g. vWF, thrombin, FIX, FXII, TSP-1, Mac-1, P-selectin), but also multiple intracellular ligands required for platelet activation [20]. The central role of the GPIb-V-IX complex in hemostasis is demonstrated by the strong bleeding disorder observed with Bernard-Soulier syndrome (BSS) patients who lack the platelet GPIb-V-IX complex, validated in studies with mice deficient in GPIbα [20, 31]. However, there are still many open questions concerning intracellular GPIb signaling and its interaction with other pathways.

When immobilized on the subendothelium of the damaged vessel wall, or presented on activated endothelial cells, the multimeric plasma protein vWF binds via its A1 domain to GPIbα and induces receptor clustering leading to platelet signaling and moderate activation. Without vessel wall components, snake venom toxins such as ristocetin or botrocetin are required for these vWF effects. Multiple intracellular signaling pathways have been proposed to mediate the intracellular effects of GPIbα activation by vWF, the phosphatidylinositol 3-kinase (PI3K)/ protein kinase B (Akt) pathway, cGMP/PKG, the mitogen-activated protein kinase (MAPK) pathways and the FcγR-Syk/PLCγ2 pathway [30, 32–36]. However, the exact mechanisms have not been fully elucidated and are controversially discussed [20, 33, 37]. GPIb-V-IX is not the only platelet vWF receptor since the integrin α_IIb_β_3_ also ligates vWF via recognition of the RGD-sequence within the C4 domain of vWF, thereby inducing also platelet activation, often together with GPIbα. As vWF is not a selective ligand of GPIbα, studies of GPIbα-selective signaling are rare and have so far been confined to platelets, which adhere to immobilized recombinant dimeric vWF A1 domain or active vWF A1-domains expressed on COS-7 cells [19, 30, 34] that also has limitations. The possible reasons for the often discrepant data and results published for GPIb signaling in human platelets are well reviewed and include the use of various GPIb-V-IX complex ligands, cell types, and biochemical and functional read-out systems [20, 38]. Recently, we developed a GPIbα-specific agonist, the C-type lectin snake venom protein, echicetin, coated on polystyrene beads [39]. Earlier studies showed that echicetin molecules cross-linked by plasma IgMκ caused platelet agglutination and weak aggregation whereas echicetin monomers, when used as specific GPIbα ligands, competed with vWF and thrombin for binding to GPIbα and blocked ristocetin/vWF mediated platelet agglutination [40]. Using a transgenic mouse model, we recently reported that the extracellular GPIbα-domain of murine platelets is essential for echicetin-bead-induced platelet aggregation [41]. In this model the extracellular domain of GPIbα is replaced by the human interleukin 4-receptor (IL4/GPIbα transgenic mice). EB induced aggregation of washed platelets from wildtype mice, but not of washed platelets expressing the IL4/GPIbα mutant protein. These data showed that EB activation of not only human but also mouse platelets requires GPIbα, the predominant receptor for most ligands of the GPIb-IX-V complex.

With these novel tools, echicetin beads (EB) and echicetin monomers (EM), we aimed to clarify whether selective activation of human platelet GPIbα has the capacity to induce activation of the tyrosine kinase Syk and whether these pathways are affected by two major platelet inhibitory pathways, cAMP/PKA and cGMP/PKG. Our results establish a novel, surprising interaction of GPIbα- and PKA/PKG-affected pathways in human platelets.

## Materials and methods

### Materials

Lyophilized snake venom of *E. carinatus sochureki* was from Latoxan, France. Lyophilized convulxin (isolated from *Crotalus durissus terrificus*) was from Enzo life sciences, Lausen, Switzerland. Human vWF isolated from plasma (Wilate®) was from Octapharma GmbH, Langenfeld, Germany. Ristocetin was from Loxo GmbH, Dossenheim, Germany. Affinity chromatography column, protein A sepharose-4B column coated with rabbit polyclonal antibodies against echicetin was from Dr. Alexei Navdaev, Würzburg, Germany. Diethylaminoethyl (DEAE) resin matrix, Toyopearls 650 S was from Tosoh Bioscience GmbH, Germany. The resin was packed in Tricorn 5/50 column from GE healthcare life sciences, Germany. Syk inhibitors, OXSI-2, 2,3-Dihydro-3-[(1-methyl-1*H*-indol-3-yl)methylene]-2-oxo-1*H*-indole-5-sulfonamide were purchased from Merck, Germany and PRT060318 (PRT-318), (2-(1R,2S)-2-aminocyclohexylamino)-4-(m-tolylamino)pyrimidine-5-carboxamide), was from Selleckchem, Germany. PP2 and its inactive analogue PP3 were from Abcam, England. Tirofiban (Aggrastat®) was from Iroko Cardio LLC, USA. MRS2179 was from Viozol, Eching, Germany, AR-C69931 from The Medicines Company, Parsippany, NJ, USA and SQ-29548, was from Cayman chemical, MI, USA. Wortmannin was purchased from Biozol, Eching, Germany. Iloprost (Ilomedine®) and riociguat were from Bayer, Germany. Bovine serum albumin (BSA) was from Capricorn Scientific GmbH, Germany. Clarity™ Western ECL Substrate was from BioRad Laboratories, USA. Rabbit monoclonal antibodies against phosphorylated Syk Y525/526 and polyclonal antibodies against phosphorylated Syk Y352, LAT Y191, PLCγ2 Y759, Akt S473, VASP S239, α-actinin were from Cell Signaling Technologies, USA. Mouse monoclonal antibodies against Syk, PLCɣ2 and Akt were from Santa Cruz Biotechnology, USA. Antibodies against β-actin were from Abcam, Engalnd. Anti- phosphotyrosine mouse antibodies were from Merck, Schwalbach, Germany. Secondary antibodies HRP-conjugated goat anti-rabbit and anti-mouse IgG were from BioRad Laboratories Hercules, USA. IP-One ELISA kit (96 wells) was from Cisbio, Codolet, France. Fluo-3, AM, intracellular cytoplasmic Ca^2+^ indicator was from Life Technologies, USA.

### Echicetin purification and echicetin-beads preparation

Echicetin was purified from *E. carinatus sochureki* lyophilized venom by affinity chromatography followed by DEAE anion exchange chromatography and validated by mass spectrometry analysis. For affinity chromatography protein A sepharose-4B column coated with rabbit polyclonal antibodies directed against echicetin (generated by A. Navdaev) was used. Echicetin was eluted using 0.2 M acetate buffer pH 2.7. The eluent buffer was exchanged into 10 mM Tris buffer pH 8.0 (buffer A) and then applied to DEAE anion exchange column. Elution of echicetin was performed by a 0 to 1 M gradient of NaCl in buffer A, under a flow rate of 1 ml/min. Fraction eluted at 120 mM NaCl consisted equally of α and β subunit and was used in all the experiments. Silver staining and mass spectrometry analysis were performed in order to confirm the purity of echicetin. Echicetin beads (EB) were prepared as reported [39] and coated for all experiments used with 0.3 mg/ml echicetin.

### LC-MS/MS

Samples from peak 1 and peak 2 were prepared under reducing conditions (by adding Laemmli buffer) then boiled at 95 °C for 10 min. Proteins of both peaks were separated by electrophoresis using 15% SDS-PAGE gels. Gels were stained using InstantBlue™. Bands were cut and digested using trypsin. Protein sequences were analyzed by mass spectrometry in the mass spectrometry core facility at the University Medical Center of the Johannes Gutenberg University, Mainz.

### Preparation of washed human platelets

Venous blood was collected as citrated whole blood after informed consent from healthy volunteers who did not take any medication for at least 10 days before blood collection. Studies using human platelets from healthy volunteers and from a patient with Glanzmann thrombosthenia caused by a homozygous point mutation in *ITGA2B* c.621C > T; p.T176I [42, 43] were approved by the local ethics committees (Study No. 837.302.12; 25.07.12; FF109/2015). EGTA (2 mM final concentration) was added to the whole blood before centrifuging at 200 x g for 10 min at room temperature (RT) to get platelet-rich plasma (PRP). PRP was diluted 1:1 with CGS buffer (120 mM NaCl, 12.9 mM Tri-Na-citrate, 30 mM glucose, pH 6.5) then centrifuged at 400 x g for 10 min at RT. The platelet pellet was resuspended with Hepes buffer (150 mM NaCl, 5 mM KCl, 1 mM MgCl_2_, 10 mM Glucose, 10 mM Hepes) pH 7.4. Agglutination followed by fibrinogen-dependent aggregation of washed human platelets was induced by adding human vWF (2.5 μg/ml final concentration) plus ristocetin (0.5 mg/ml final concentration) as previously described [41]. Residual platelet aggregation was calculated by subtraction of overall maximum light transmission in the absence of tirofiban minus maximum light transmission in the presence of tirofiban. Washed platelets were placed for 20 min at 37 °C for resting. Human washed platelets (3 × 10^8^ platelets/ml) were pre-incubated at 37 °C with different inhibitors: Syk inhibitors, OXSI-2 or PRT-318, or Src family kinase inhibitor PP2 or its inactive analogue PP3 or wortmannin. In addition, ADP and TxA2 receptors blockers were used (AR-C69931, MRS2179, SQ-29548), cAMP-elevating agents iloprost and cGMP-elevating soluble guanylate cyclase (sGC) stimulator riociguat, respectively. Platelet aggregation was triggered with 0.5% (v/v) echicetin beads for 1, 2 and 5 min under continuous stirring (1000 rpm) at 37 °C in an Apact4S Plus aggregometer (DiaSys Greiner, Flacht, Germany). Control samples mentioned as basal (in graphs) or time zero (in blots and graphs) refer to washed platelets placed under continuous stirring for 1 min in absence of any agonist.

### Western blot analysis

Western blot samples were prepared by immediately adding Laemmli buffer to washed platelets inside the cuvettes and boiled at 95 °C for 10 min. Platelet proteins were separated by electrophoresis using 8% SDS-PAGE gels then transferred to polyvinylidene difluoride *(*PVDF*)* membranes before blocking the membranes for one hour with 5% BSA in TBS-T (20 mM Tris, 140 mM NaCl, 0.1% Tween, pH 7.6) at room temperature. Membranes were incubated overnight under gentle shaking with 1:1000 diluted primary antibodies. Membranes were washed three times with TBS-T and incubated for two hours at RT with the appropriate secondary antibodies diluted 1:5000 in 5% BSA. Membranes were washed again for 3 times with TBS-T before developing the blots by ECL detection. Control blots with total proteins (Syk, PLCγ2 and Akt) derive from same samples from the corresponding phosphorylated form. α-actinin was also used as loading control for distinct p-Akt blots. Blots with p-LAT, p-VASP were stripped and reprobed with anti-β-actin antibody. Blots were cropped following the corresponding molecular weight of the protein of interest.

### Inositol monophosphate (InsP1) measurement

The production of inositol triphosphate (InsP3) was measured by the level of the InsP1 accumulated in washed platelets using the IP-One ELISA kit according to the manufacturer’s instructions. Human washed platelets adjusted to 3 × 10^8^/ml in presence of LiCl (1 mM), inhibiting the degradation of InsP1 into *myo*-inositol. Washed platelets were activated by EB in the absence or presence of effectors, under stirring conditions. Platelets were lysed after 5 min of stimulation then centrifuged at 16,000 x g for 10 min at 4 °C. Platelets lysates were incubated with InsP1- HRP conjugate and anti- InsP1 monoclonal antibody for 3 h.

### Intracellular Ca^2+^-release measurement

Human platelets were prepared as already mentioned above and adjusted to 3 × 10^8^/ml in Hepes buffer. Washed platelets were then pre-incubated with a Ca^2+^ indicator dye, fluo-3 acetoxymethyl (AM) esters (5 μM) for 30 min at 37 °C. Intracellular Ca^2+^-release was monitored for 2 min by flow cytometry after stimulation with EB without additional supplementation of extracellular Ca^2+^.

### Statistical analysis

Experiments were performed at least three times with at least three different healthy donors. The data were presented as mean ± standard deviation (S.D.). Statistical analysis was performed using GraphPad Prism 7 for Windows (GraphPad Software, San Diego, CA). The 2-tailed Student’s t-test was used for comparison of two groups, one-way and two-way ANOVA and Tukey’s multiple comparison tests were used, when appropriate, for comparison of more than two groups. *P* < 0.05 was considered as significant.

## Results

### Echicetin coated polystyrene beads activate Syk tyrosine kinase and stimulate α_IIb_β3 integrin-dependent platelet aggregation

Initial experiments with conventionally purified echicetin from the snake venom *Echis carinatus sochureki* produced variable effects on platelets and prompted us to modify the purification procedure (Additional file 1: Figure S1). The final DEAE column produced 2 major protein peaks, which were characterized by mass spectrometry. Only peak 1 contained pure echicetin heterodimer (with α + β subunits), whereas peak 2 contained primarily the ß subunit and some unidentified bands (Additional file 1: Figure S1A, B). Echicetin eluted in peak 1 was used for all experiments reported here with consistent results, both with echicetin-coated beads (EB) or as echicetin monomer (α/ß subunits) (EM).

In agreement with our earlier work [39], the α_IIb_β3 integrin antagonist tirofiban completely inhibited EB-induced platelet aggregation of washed platelets. EM, which alone selectively bind to GPIbα without inducing platelet activation, completely inhibited EB-induced platelet aggregation in a competitive manner. As negative-control, BSA coated beads did not show any effect on platelet aggregation (Additional file 1: Figure S1C, D). We also tested whether EM affect platelet aggregation induced by other main platelet receptors. Echicetin did not affect the aggregation induced by collagen (Additional file 2: Figure S2A, B), ADP (Additional file 2: Figure S2C, D), TxA_2_ (Additional file 2: Figure S2E, F), TRAP6 (Additional file 2: Figure S2G, H), thrombin at high concentration (Additional file 2: Figure S2I, J). A small inhibitory effect was observed with aggregation induced by a low concentration of thrombin (Additional file 2: Fig. S2K, L), which possibly reflects the thrombin binding site of GPIbα [20, 44].

**Fig. 1:**
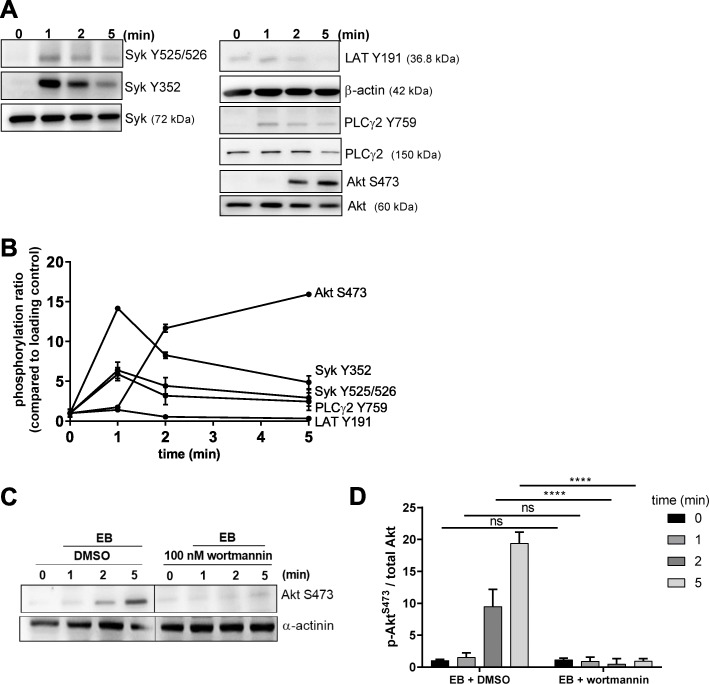
Echicetin-coated polystyrene beads activate Syk tyrosine kinase and the PI3K-dependent serine/threonine kinase Akt. **a** Human washed platelets (WP) were stimulated under stirring conditions with EB . Platelet aggregation (see Fig. S1C, D) was stopped after 1, 2 or 5 min by adding Laemmli buffer. Tyrosine phosphorylation of Syk, LAT, PLCγ2 and serine phosphorylation of Akt were analyzed by immunoblotting compared to the total protein respectively. **b** The kinetics of the phosphorylation patterns represent means ± S.D. of 3 independent experiments with platelets from 3 healthy donors. **c** WP were pre-incubated for 5 min at 37 °C with vehicle control (DMSO) or with the PI3K inhibitor wortmannin (100 nM final concentration) prior to stimulation with EB. **d** Quantitative data of Akt S473 phosphorylation compared to the loading control α-actinin are represented as means ± S.D. of 3 independent experiments with platelets from 3 healthy donors (Samples were loaded on the same gel; a black line was used to indicate that a group of samples not related to this data set was not shown); ns: not significant, ****p < 0.0001

For the possible Syk activation by EB, we analyzed the phosphorylation kinetics of the Syk activation marker, tyrosine 525/526, which is located in the activation loop of the kinase domain [45]. Washed human platelets stimulated with EB showed increased, but transient Syk phosphorylation at Y525/526 in a time dependent manner. EB-induced Syk Y526 phosphorylation was confirmed by LC-MS/MS (data not shown). Additionally, we investigated the phosphorylation of Syk at Y352, which is located in the SH2 kinase linker domain and shows an important role in Syk activation [23, 46]. Furthermore, we also measured the tyrosine phosphorylation of direct Syk substrates, inker for activation of T-cells (LAT) and phospholipase Cγ2 (PLCγ2), as indicator of Syk kinase activity. LAT Y191 phosphorylation was detectable but weak whereas PLCγ2 Y759 phosphorylation had a similar time course as Syk tyrosine phosphorylation. In contrast, one of Syk downstream effectors and important PI3K effector, Akt, showed a delayed phosphorylation at S473 (Fig. 1a, b), which was completely inhibited by the PI3K inhibitor wortmannin (Fig. 1c, d), showing PI3K-dependency of Akt phosphorylation in EB-induced GPIbα signaling.

### EB-induced Syk tyrosine phosphorylation requires src family kinases (SFKs)

**Fig. 2:**
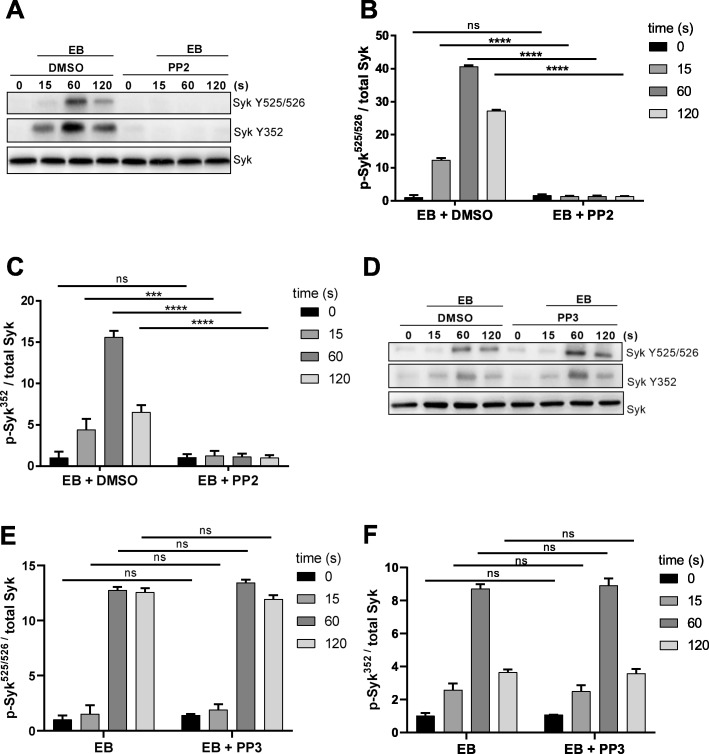
EB-induced Syk tyrosine phosphorylation is dependent on src family kinases (SFKs). Human washed platelets (WP) were pre-incubated for 5 min with vehicle control (DMSO), the SFK inhibitor, PP2 (10 μM) or with its inactive analogue, PP3 (10 μM) prior to stimulation with EB. **a**, **d** Syk phosphorylation at Y525/526 and Y352 was analyzed at early time points (0, 15, 60 and 120 s). **b**, **c**, **e**, **f** Quantitative analysis of Syk Y525/526 and Y352 phosphorylation compared to total Syk. Data are means ± S.D. from at least 3 independent experiments with platelets from at least 3 healthy donors; ns: not significant, ***p < 0.001, ****p < 0.0001

In order to study the role of SFKs in Syk activation, washed platelets were pre-incubated with a vehicle control (DMSO), with the SFK inhibitor PP2 and its inactive analogue PP3. EB-induced platelet aggregation was strongly inhibited only in the presence of PP2 (Additional file 3: Figure S3A, B). PP2 abolished time-dependently (0, 15, 60 and 120 s) EB-induced Syk phosphorylation at Y525/526 and Y352 (Fig. 2a, b, c). In contrast, neither Syk Y525/526 phosphorylation (Fig. 2d, e) nor Syk Y352 phosphorylation (Fig. 2d, f) were inhibited by PP3. Interestingly, dasatanib (100 nM) also inhibited EB-induced platelet aggregation and EB-induced Syk Y352 and Y525/526 phosphorylation (data not shown). Dasatinib, originally developed for the treatment of chronic myelogenous leukemia (CML), is not only an inhibitor of BCR-ABL but also a potent inhibitor of SFKs [47].

### Syk inhibitors demonstrate an essential role of Syk in EB-induced platelet aggregation and a differential regulation of Syk Y525/526 and Y352 phosphorylation

**Fig. 3:**
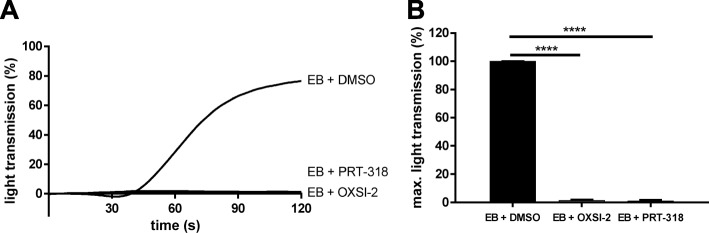
Syk-inhibitors strongly block EB-induced platelet aggregation. Human washed platelets (WP) were pre-incubated with 2 different Syk inhibitors, OXSI-2 (2 μM) and PRT-318 (1 μM) for 5 min prior to stimulation with EB. **a** Representative curves showing the effect of Syk inhibitors on platelet aggregation and **b** the corresponding quantification of data as means ± S.D. from 3 independent experiments with platelets from 3 healthy donors; ****p < 0.0001

**Fig. 4:**
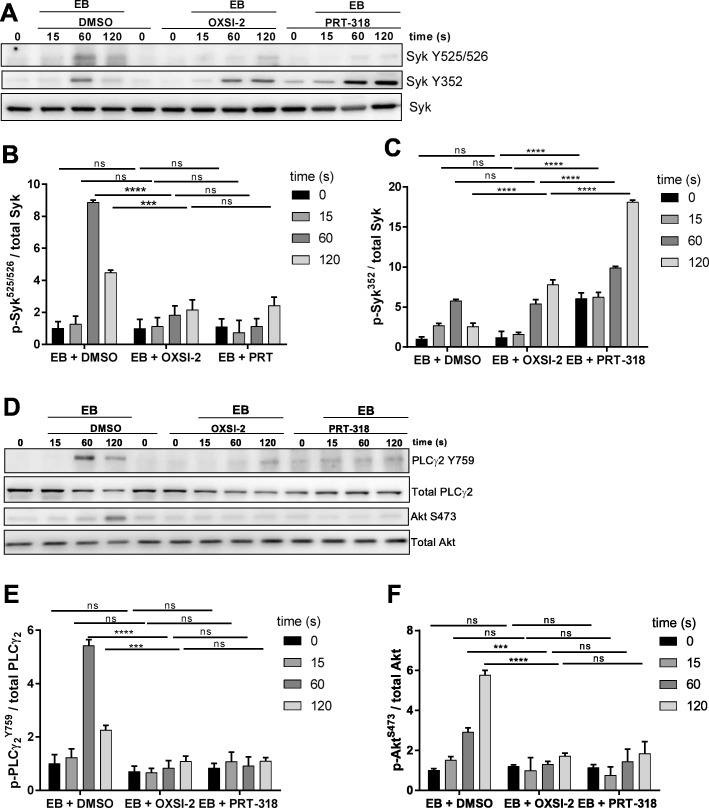
Syk inhibitors differentially affect Syk tyrosine phosphorylation. Human washed platelets (WP) were treated as mentioned in Fig. 3. Samples were taken after 15, 60 and 120 s**. a** Representative blots of Syk phosphorylation on Y525/526 and Y352 and the corresponding quantification shown as ratio compared to the total Syk protein represented in **b** and **c** respectively. **d** Representative blots of Syk downstream effectors PLCγ2 Y759 and Akt S473 and the corresponding quantification **e** and **f** shown as ratio compared to the total PLCγ2 and Akt respectively. Data are means ± S.D. from 3 independent experiments with platelets from 3 healthy donors; ns: not significant, ***p < 0.001, ****p < 0.0001

To investigate the role of Syk in mediating EB- induced platelet activation, platelets were pre-incubated with two different, well-described Syk inhibitors, OXSI-2 or PRT-318 [48–50]. Platelet aggregation was completely abolished by those two inhibitors (Fig. 3a, b), and global EB-induced tyrosine phosphorylation was partially inhibited (Additional file 4: Figure S4). Both Syk inhibitors caused a complete inhibition of EB-stimulated Syk Y525/526 phosphorylation, whereas Syk Y352 phosphorylation was not inhibited but prolonged (with OXSI-2) and prolonged/enhanced with PRT-318 (Fig. 4a, b, c), which clearly differed from the PP2 effects. These Syk inhibitors also strongly reduced EB-induced phosphorylation of PLCγ2 at Y759, a direct Syk downstream target, and Akt at S473, a probably indirect Syk downstream target (Fig. 4d, e, f). For Akt we could show that the PI3K inhibitor wortmannin abolished EB-mediated Akt S473 phosphorylation (Fig. 1c, d) without inhibition of Syk tyrosine phosphorylation (data not shown). To compare these EB-mediated platelet effects with the classical platelet GPIbα-agonist ristocetin, we analyzed vWF/ristocetin-induced aggregation and Syk phosphorylation of washed human platelets in the presence of the Syk inhibitor PRT-318. PRT-318 inhibited partially vWF/ristocetin-induced platelet aggregation, but tirofiban-mediated inhibition of platelet aggregation was not further diminished by the PRT-318 compound (Additional file 4: Figure S4B, C), indicating that only vWF-mediated platelet aggregation but not agglutination was affected by the Syk inhibitor. Whereas vWF/ristocetin-induced Syk Y525/526 phosphorylation was clearly inhibited by PRT-318 (Additional file 4: Figure S4D), the effects were not markedly different in the presence of tirofiban. As observed for EB also vWF/ristocetin-induced Syk Y352 phoshorylation was not affected by PRT-318. To confirm that vWF/ristocetin-mediated Syk activity was inhibited by PRT-318, LAT was studied as direct substrate of Syk. vWF/ristocetin-induced LAT Y191 phosphorylation was similarly induced by vWF/ristocetin in the absence or presence of tirofiban and downregulated to basal levels in the presence of PRT-318 (Additional file 4: Figure S4, E). In addition, vWF/ristocetin-stimulated Akt S473 phosphorylation was downregulated by Syk inhibition, too. However, in the presence of tirofiban Akt phosphorylation was more diminished, indicating a role of integrin α_IIb_β_3_ outside-in signaling in Akt S473 phosphorylation induced by vWF/ristocetin (Additional file 4: Figure S4E). These data demonstrate that Syk plays an important role in GPIbα-mediated platelet activation induced by EB as well as by vWF/ristocetin.

### EB-induced platelet aggregation requires the secondary mediators ADP and TxA_2_

**Fig. 5:**
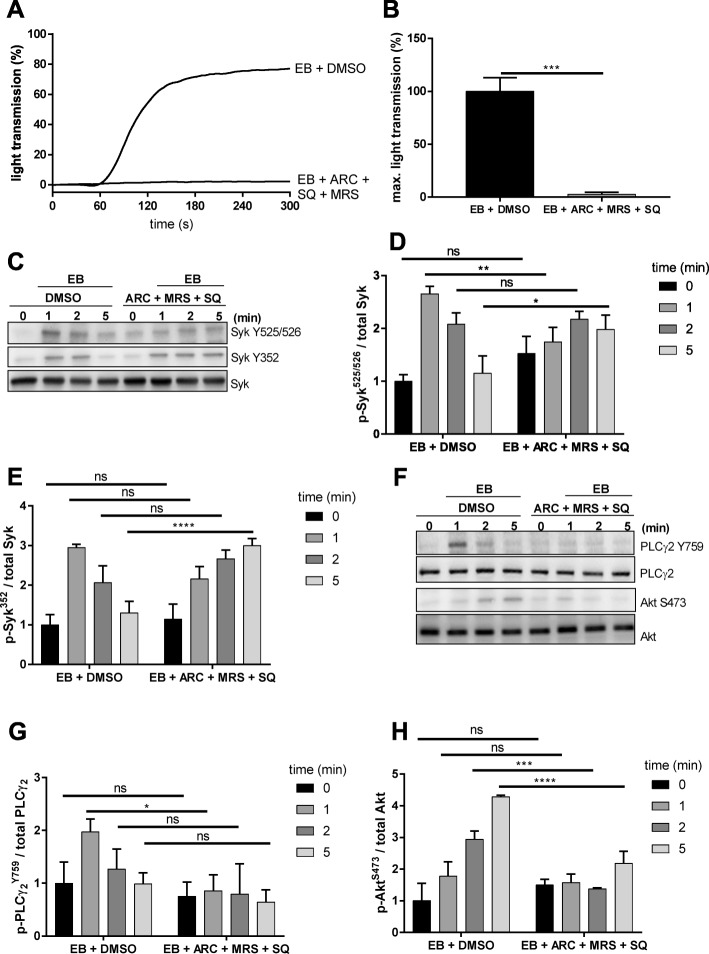
ADP and TxA_2_ regulate EB-induced platelet aggregation and Syk downstream signaling. Human washed platelets (WP) were pre-incubated for 5 min simultaneously with P2Y_12_ antagonist, AR-C69931 (0.1 μM), P2Y_1_ antagonist, MRS2179 (1 μM) and TxA_2_ receptor antagonist, SQ-29548 (1 μM) prior to stimulation with EB. Samples were taken after 1, 2 and 5 min. **a** Representative curves of platelet aggregation mediated by EB in the presence of the vehicle control or the 3 inhibitors and the corresponding quantification are represented in **b**. **c** Representative blots of Syk phosphorylation on Y525/526 and Y352 and the corresponding quantification shown as ratio compared to the total Syk protein represented in **d** and **e** respectively. **f** Representative blots of Syk downstream effectors PLCγ2 Y759 and Akt S473 and the corresponding quantification **g** and **h** shown as ratio compared to the total PLCγ2 and Akt, respectively. Data are presented as means ± S.D. from 3 independent experiments with 3 healthy donors; ns: not significant, *p < 0.1, **p < 0.01, ***p < 0.001, ****p < 0.0001

Platelet adhesion receptor pathways variably require the activation-induced release of secondary mediators such as ADP and TxA_2_ for a full response [10]. Therefore, we evaluated the involvement of ADP and TxA_2_ in EB-induced platelet aggregation and phosphorylation responses by simultaneously blocking the P2Y_12,_ P2Y_1,_ and TP receptors using AR-C69931, MRS2179 and SQ-29548, respectively. These conditions completely prevented platelet aggregation (Fig. 5a, b), whereas EB-induced Syk Y352 phosphorylation was not inhibited but instead prolonged at late time points (Fig. 5c, d, e). EB-induced Syk Y525/526 phosphorylation was partially inhibited by these compounds. In addition, phosphorylation of the direct downstream Syk effector, PLCγ2 Y759, as well phosphorylation of Akt at S473 (Fig. 5f, g, h) were significantly inhibited.

### EB-mediated Syk activation does not require integrin α_IIb_β_3_ signaling

**Fig. 6:**
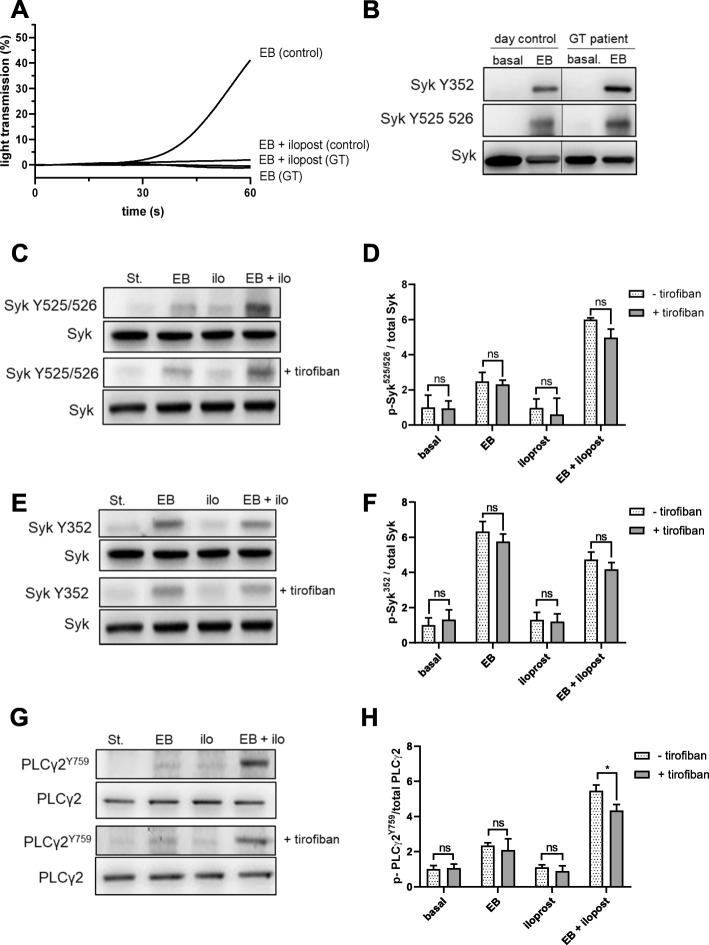
EB-induced Syk activation is not dependent on integrin αIIbβ3 outside-in signaling in contrast to the overall platelet aggregation. **a** Washed platelets (WP) from a patient with Glanzmann thrombasthenia (GT) and from a healthy donor (day control) were pre-incubated at 37 °C for 3 min in the presence or absence of 2 nM iloprost prior to stimulation with EB. Platelet aggregation was monitored until 60 s using light transmission aggregometry under stirring conditions and then stopped using Laemmli buffer for immunoblotting. **b** Platelet aggregation was stopped after 60 s using Laemmli buffer and Syk Y525/526 and Y352 were analyzed by immunoblotting. WP from a healthy donor were pre-incubated with iloprost (2 nM, 3 min) in the presence or absence of tirofiban (1.25 μg/ml, 1 min) prior to stimulation with EB. Platelet aggregation was stopped after 60 s using Laemmli buffer. Phosphorylation of **c** Syk Y525/526, **e** Syk Y352 and **g** PLCγ2 Y759 were analyzed by western blot. Quantification of p-Syk **d** Y525/526 and **f** Y352 compared to total Syk and **h** PLCγ2 Y759 compared to total PLCγ2. Data are from 3 different experiments with platelets from 3 healthy volunteers and presented as means ± S.D. ns: not significant, *p < 0.05

Many signaling platelet pathways are affected by integrin out-side-in signaling, especially by the integrin α_IIb_β_3_. To address the possible role of the integrin α_IIb_β_3_ for GPIbα-induced Syk activation we studied washed platelets from a patient with Glanzmann thrombasthenia (GT), which showed severe reduction of the major fibrinogen receptor α_IIb_ß_3_ and which has been studied previously [51]. As expected, EB did not induce any aggregation response of platelets from a patient with GT, whereas platelets from a healthy control showed clear aggregation in response to EB, which was completely inhibited by the cAMP-elevating agent iloprost (Fig. 6a). EB induced the phosphorylation of Syk at both sites Y525/526 and Y352 in thromboasthenic as well as in control platelets (Fig. 6b). Furthermore, we investigated the effect of the integrin α_IIb_β_3_ inhibitor tirofiban on Syk activation with platelets from healthy controls. EB-induced Syk phosphorylation in platelets pre-incubated with tirofiban was not different when compared to non-treated control platelets. Also, the combination of iloprost and tirofiban did not differ in the phosphorylation pattern of Syk compared to platelets pre-incubated with iloprost alone (Fig. 6c, d, e, f). Additionally, tirofiban alone did not inhibit the phosphorylation of PLCγ2 Y759. Only the combination, tirofiban plus iloprost, induced a partial inhibition in the phosphorylation pattern (Fig. 6 g, h). The effects of iloprost alone on EB-induced Syk phosphorylation are presented next.

### cAMP/ cGMP elevation inhibits EB-induced platelet aggregation but not Syk activation, an effect also observed with convulxin-treated platelets

**Fig. 7:**
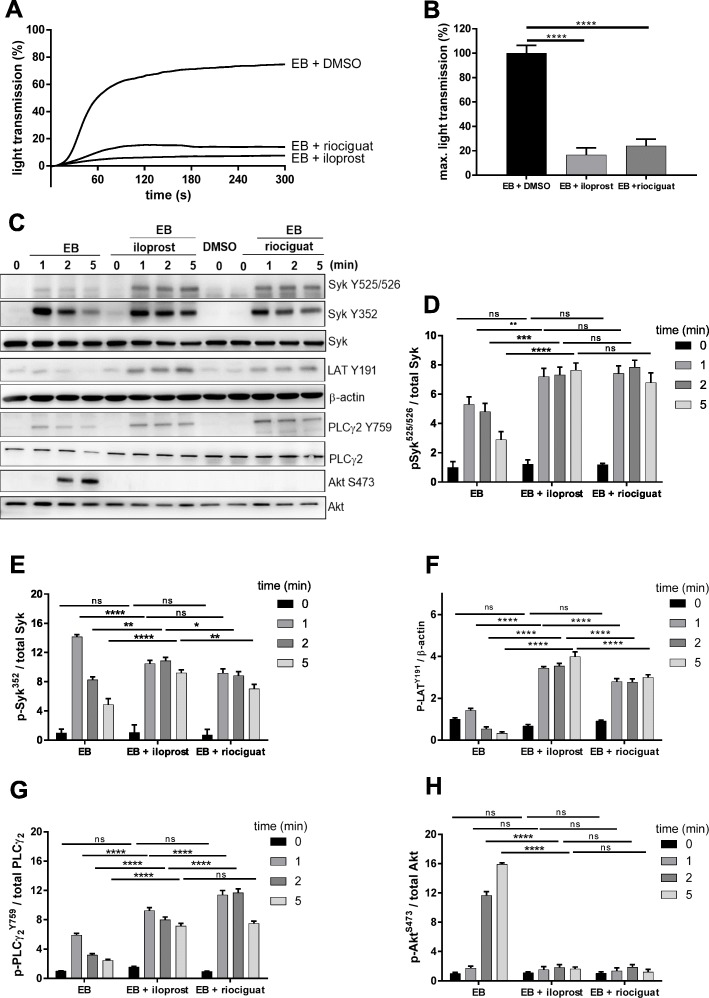
Iloprost and riociguat inhibit EB-induced platelet aggregation but do not inhibit tyrosine phosphorylation of Syk. Washed platelets (WP) were pre-incubated for 3 or 2 min with 2 nM iloprost or 20 μM riociguat respectively prior to stimulation with EB. **a** Aggregation curves and the corresponding quantitative data are shown in **b**. **c** Aggregation was stopped after 1, 2 or 5 min using Laemmli buffer. Syk Y525/526 and Y352, Syk downstream effectors LAT Y191, PLCγ2 Y759 and Akt S473 were analyzed by western blot. **d**, **e**, **f**, **g**, **h** Quantification of the phosphorylated proteins are represented as ratio compared to the total protein or compared to β-actin for LAT analysis. Results are presented of least 3 different experiments with platelets from at least 3 healthy volunteers, data are represented as means ± S.D. ns: not significant, *p < 0.1, **p < 0.01, ***p < 0.001, ****p < 0.0001

We then evaluated possible effects of the cAMP/PKA and cGMP/PKG pathways on GPIbα-mediated platelet signaling and aggregation. Previously, we had established conditions for specific cAMP/PKA- or cGMP/PKG-mediated phosphorylation of established PKA and/or PKG substrates (e.g. VASP S157, VASP S239, CALDAG GEF1 S587, phosphodiestersase PDE5A S102) by functional studies, immunoblotting and phosphoproteomic analysis using iloprost or riociguat [8, 52–54]. Here, pre-incubation of washed platelets with increasing concentrations of iloprost (Additional file 5: Figure S5A) and riociguat (Additional file 5: Figure S5B) inhibited EB-stimulated platelet aggregation in a dose dependent manner. For further studies, we used 2 nM iloprost and 20 μM riociguat (with established phosphoproteomic responses [8, 52]), which caused a strong inhibition of EB-stimulated platelet aggregation (Fig. 7a, b). Under these conditions, a consistently robust and stable phosphorylation of VASP S157 (substrate for PKA > PKG) and VASP S239 (substrate for PKG > PKA) was observed. Whereas VASP 239 phosphorylation was measured by phosphoantibodies, VASP S157 phosphorylation is indicated by the apparent shift of VASP from the 46 kDa to the 50 kDa form in SDS PAGE. Here, it is important to note that EB alone did not result in any VASP phosphorylation and that the observed iloprost or riociguat-stimulated VASP phosphorylation was not down-regulated by EB treatment (Additional file 5: Figure S5C, D). Furthermore, the PKG specific substrate phosphodiesterase PDE5A S102 was phosphorylated only in the riociguat-treated samples, but not in response to iloprost (data not shown). Overall, our present results show the selective and robust activation of PKA and PKG by iloprost and riociguat, respectively. Then, the effects of PKA or PKG pathways on the kinetics of the EB-induced Syk activation and phosphorylation of selected downstream effectors (LAT, PLCγ2 and Akt) were investigated. In contrast to the total inhibition of EB-induced aggregation, iloprost and riociguat did not inhibit but enhanced/prolonged phosphorylation of the Syk activation marker Y525/526 (hyperphosphorylation), detectable at early and late time points of EB stimulation (Fig. 7c, d). With the regulatory site Y352, iloprost and riociguat moderately reduced phosphorylation initially (at 1 min EB stimulation) but then (2 min, 5 min) did not inhibit but prolong Syk Y352 phosphorylation (Fig. 7c, e). Since the data indicate that the strong inhibitory pathways PKA/PKG do no prevent Syk activation under these conditions we sought to confirm this at the level of the Syk substrates LAT Y191 (Fig. 7c, f) and PLCγ2 Y759 (Fig. 7c, g). Both Syk-mediated phosphorylation events were not inhibited but strongly enhanced/prolonged until 5 min of activation. In contrast, the EB-induced, delayed Akt phosphorylation at S473 was abolished by both iloprost and riociguat (Fig. 7c, h), which resembles the aggregation response.

Then we also used lower concentrations of iloprost (1 nM) and riociguat (10 μM), which partially inhibit EB-induced platelet aggregation, to test if the observed protein phosphorylation effects are still present compared to 2 nM iloprost and 20 μM riociguat, respectively. For 1 nM iloprost (Additional file 6: Figure S6A-E) as well as for 10 μM riociguat (Additional file 7: Figure S7A-E) we observed also increased and prolonged phosphorylation of Syk Y525/526, Syk Y325 and PLCγ2 Y759 as well as diminished Akt S473 phosphorylation (Additional file 6: Figure S6D, F and Additional file 7: Figure S7D, F), indicating similar effects of iloprost and riociguat on EB-induced platelet signaling.

The surprising differential effects of the inhibitory pathways (PKA/PKG) on EB-simulated platelet aggregation and Syk activation prompted us to study also a classical mechanism of Syk activation in human platelets, the convulxin/GPVI pathway [10–12]. For the analysis of PKA/PKG effects on GPVI-mediated signaling pathways, we used 50 ng/ml convulxin to stimulate washed human platelets. Convulxin-stimulated platelet aggregation was completely inhibited by iloprost and riociguat (Additional file 8: Figure S8A, B). Then, convulxin-induced Syk phosphorylation in the presence of a vehicle control, iloprost or riociguat was analyzed. The Syk activation marker Y525/526 and the regulatory site Y352 were well but only transiently phosphorylated in response to convulxin (Additional file 8: Figure S8C). Strikingly, convulxin-stimulated Syk Y525/526 phosphorylation was significantly increased/prolonged (hyperphosphorylation) by iloprost and riociguat compared to the vehicle control (Additional file 8: Figure S8C, D), which was supported by LC-MS/MS (data not shown). With Syk Y352 phosphorylation, there was little effect after 1 min of cvx stimulation, but at 2 and 5 min there was also significantly increased and prolonged phosphorylation in the presence of both inhibitory agents (Additional file 8: Figure S8C, E).

### EB-stimulated intracellular InsP1 accumulation and Ca^2+^-release are only partially inhibited by iloprost and riociguat

**Fig. 8:**
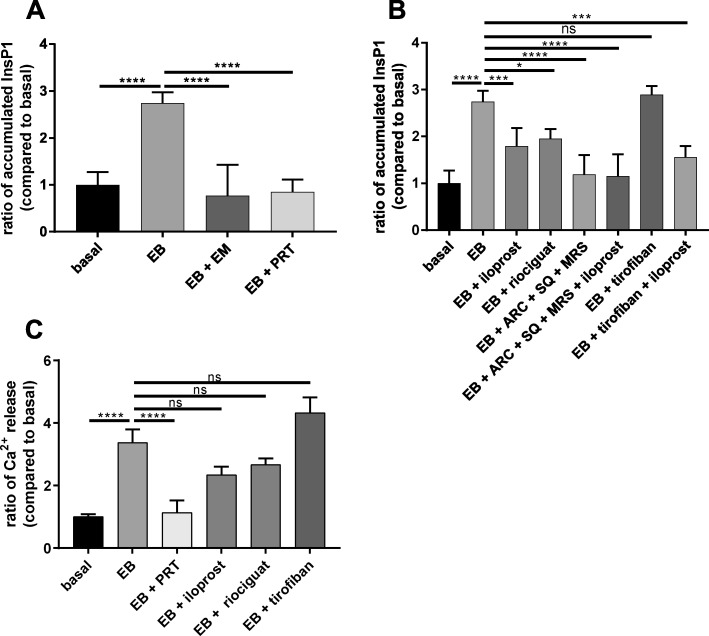
Differential regulation of InsP1 accumulation and Ca^2+^-release induced by EB. InsP1 accumulation was measured due to InsP3 production. **a**, **b** Human washed platelets (WP) were pre-incubated as described before with the various reagents used in the previous experiments prior to stimulation with EB in presence of 1 mM LiCl. Aggregation was stopped after 5 min using the lysis buffer provided by the manufacturer. **c** WP preincubated with Fluo-3, AM (5 μM) for 30 min at 37 °C before treating platelets. Ca^2+^-release was monitored for 120 s using flow cytometry. Data are representative of 3 different experiments from 3 healthy donors. Results are means ± S.D. ns: not significant, *p < 0.1, ***p < 0.001, ****p < 0.0001

In order to analyze a functional response in platelets directly after Syk–mediated PLCγ2 phosphorylation/activation, we measured EB-induced InsP1 accumulation (in the presence of lithium) as marker of EB-mediated InsP3 production as reported [55]. We detected a clear 3-fold increase of InsP1 accumulation of EB-treated platelets, which was abolished by pre-incubation with EM (as control) and by the Syk inhibitor PRT-318 (Fig. 8a). Iloprost and riociguat only partially inhibited this EB-mediated InsP1 accumulation, which was more strongly reduced when ADP/TP receptors were blocked. The combination of these inhibitors did not produce additive effects (Fig. 8b). Also, pre-incubation of platelets with tirofiban did not reduce InsP1 accumulation, and the combination of iloprost with tirofiban showed the inhibition of EB-induced InsP1 production as with iloprost alone (Fig. 8b). Furthermore, we detected a significant increase of endogenous Ca^2+^-release upon platelet activation with EB, which was abolished by PRT-318. However, iloprost and riociguat showed only partial inhibition of EB-induced Ca^2+^-release. In addition, EB-induced Ca^2+^-release showed to be independent from the integrin α_IIb_ β_3_ outside-in signaling as tirofiban had no significant effect (Fig. 8c).

## Discussion

In this study we demonstrated that cAMP/PKA and cGMP/PKG pathways cause dichotomous regulation of GPIbα-mediated Syk stimulation and activation of human platelets. To ensure selective GPIbα-activation the specific GPIbα-ligand echicetin was used as multimer complex, coated on polystyrene beads (EB), leading to integrin αIIbβ3-dependent aggregation of human platelets [39–41]. Echicetin monomer (EM) antagonized all observed EB effects but not GPVI/FcRγ or GPCR-mediated platelet aggregation.

EB induced platelet Syk phosphorylation and aggregation, which requires SFKs as these effects are prevented by the SFK inhibitors PP2 and dasatinib. Syk activation by EB in human platelets as assessed by Y352 and Y525/526 phosphorylation, was rapid but transient, indicating substantial dephosphorylation. Interestingly, dephosphorylation of murine Syk at Y346 (~ Y352 in the human kinase) by the TULA-2 protein-tyrosine phosphatase suppressed its activation in murine platelets [56].

As reported by us recently, EB strongly activated murine platelets, expressing only FcRγ-chain but not FcRγIIA, which required GPIbα [41]. Our previous quantitative proteomic studies with human platelets demonstrated expression of FCER1G (8170 copies), FCGR2A (990 copies) and Syk (4900 copies) [57]. Within a major, still ongoing phosphoproteomic study (to be published) we recently detected multiple tyrosine-phosphorylated proteins in EB-stimulated human platelets including dually Y-phosphorylated FCER1G (Y56 and Y76, both 1.8 fold increased phosphorylation). Tyrosine phosphorylation of FCGR2A was not detected.

Altogether, the data indicate that EB activation of the human platelet GPIb-complex stimulates Syk phosphorylation via an SFK-dependent mechanism, which is antagonized by EM. SFK-dependent Syk activation involves direct Syk Y352 phosphorylation and may also involve the platelet ITAM protein FcRγ-chain.

To validate a functional role of Syk for GPIbα-signaling in human platelets, we tested two different Syk inhibitors, OXSI-2 and PRT-318. These have been described as Syk and platelet function inhibitors in studies of convulxin/GPVI signaling [48–50]. PRT-318 also prevented heparin-induced thrombocytopenia (HIT) and thrombosis in a mouse model [49]. Another novel orally bioavailable Syk inhibitor (BI1002494) prevented arterial thrombosis and thromboinflammatory brain infarction in a mouse model to a similar extent as did, in another mouse model, platelet-specific Syk deficiency [58]. In humans, the Syk inhibitor fostamatinib was recently FDA-approved for the treatment of thrombocytopenia in adult patients with chronic immune thrombocytopenia (ITP) [59].

OXSI-2 (2 μM) and PRT-318 (1 μM) abolished EB-induced aggregation and strongly inhibited EB–induced Syk Y525/526, but not Syk Y352, phosphorylation. These inhibitors also prevented EB-induced phosphorylation of the direct Syk substrate (PLCγ2 at Y759) and a further downstream effector, Akt S473. Syk Y525/526 phosphorylation, primarily due to autophosphorylation [23, 24], is expected to be blocked by Syk inhibitors. In contrast, Syk Y348/Y352 phosphorylation catalyzed by SFKs in intact cells initiates Syk activation [23], and is not blocked by Syk inhibitors but by the SFK inhibitor PP2, as observed here in our studies. Absent inhibition of platelet agonist–induced Syk Y348/Y352 phosphorylation, catalyzed by SFKs, has been used as one specificity criteria for Syk inhibitors [48, 49]. For the overall regulation/activation of Syk, the sites Y348/Y352 are considered more important than the activation loop sites Y525/Y526 since mutations of latter sites did not reduce Syk kinase activity [23, 24]. This indicates that Syk tyrosine phosphorylation alone cannot be equated with Syk kinase activity. The strong inhibitory effects of Syk inhibitors on EB-induced Syk Y525/526, PLCγ2 Y759 phosphorylation, and EB-induced aggregation, indicate that EB activate human platelets by a Syk-dependent process.

Based on the inhibitory effects of the Syk inhibitors, EB-induced Akt phosphorylation requires Syk and therefore appears to be downstream of Syk activity. However, Akt is certainly not an immediate Syk target/direct substrate but is most likely phosphorylated in response to additional adapter proteins affecting PI3K [10], which is confirmed by our results that the PI3K inhibitor wortmannin abolished EB-mediated Akt S473 phosphorylation without inhibition of Syk tyrosine phosphorylation.

A recent paper reported that Syk activity is dispensable for platelet GPIb-IX-V signaling induced by ristocetin/vWF [37] by showing unaffected phosphorylation of Syk at Y352 and of Akt at S473 by the Syk inhibitor PRT-318. Using the same Syk inhibitor we confirmed that ristocetin/vWF-induced Syk Y352 phosphorylation is not affected. However, we could show that PRT-318 inhibited phosphorylation of Syk at Y525/526, of the Syk substrate LAT at Y191 and of Akt at S473 in washed human platelets, indicating that Syk plays an important role in EB- as well in ristocetin/vWF-mediated GPIbα-signaling of human platelets.

The receptors GPVI and CLEC-2 activate platelets by a Syk-dependent mechanism and require the release of secondary mediators (ADP,TxA_2_) for a full response, with certain differences [10]. Therefore, the role of the secondary mediators for the EB responses studied here was investigated. EB–induced platelet aggregation was abolished when the ADP receptors (P2Y_12_ and P2Y_1_) and the TxA_2_ receptor were blocked. In contrast, Syk phosphorylation was not at all (Y352) or only partially (Y525/Y526) reduced, whereas PLCγ2 Y759 and Akt S473 phosphorylation was strongly reduced. We have no clear explanation for the partial inhibition of Syk 526/526 phosphorylation by the secondary mediators except to speculate that this site may also be affected by pathways other than SFKs. This has also been proposed for PLCγ2 and its phosphorylation [35]. In our platelet phosphoproteomic studies, ADP only stimulated Syk serine phosphorylation with no detectable effect on Syk tyrosine phosphorylation [53], which was confirmed in our ongoing experiments (Makhoul S et al., unpublished data). Overall, our data show that ADP and TxA2 have no major effect on EB-induced Syk phosphorylation but, in contrast, are required for further downstream effects including PLCγ2 Y759/Akt S473 phosphorylation and aggregation.

In addition to ADP and TxA_2_, other pathways such as integrin α_IIb_β_3_ activation influence various steps of platelet activation including ITAM (FcγRIIA)-dependent Syk stimulation [18, 60]. To evaluate the possible contribution of α_IIb_β_3_ for EB-induced signaling, we compared platelets from normal controls and α_IIb_β_3_-deficient platelets from a patient with Glanzmann thrombasthenia. There was no difference between normal and α_IIb_β_3_-deficient platelets with respect to EB-induced Syk activation as indicated by Y352 and Y525/Y526 phosphorylation. Similarly, the α_IIb_β_3_ inhibitor tirofiban did not affect EB-induced Syk tyrosine phosphorylation and phosphorylation of the Syk substrate PLCγ2 at Y759. These data show that EB/GPIb-mediated phosphorylation and activation of Syk is integrin α_IIb_β_3_-independent.

Platelet functions are tightly regulated by a network of intracellular pathways consisting of protein kinases/protein phosphatases and their substrates [6, 8, 61], but the regulation of specific GPIb-mediated signaling in human platelets by the PKA/PKG inhibitory pathways is unclear. PKA-stimulated GPIbß phosphorylation (S166, now S191) has been observed in multiple studies with human platelets [52, 62, 63]. When tested in the Chinese hamster ovary cell (CHO), PKA phosphorylation of GPIbß S166 correlated with reduced binding of vWF to the GPIb-IX complex [62] whereas other studies did not observe a functional effect of this phosphosite [63]. Clearly, PKA phosphorylation of GPIbß S166 (S191) and of other sites within the GPIb-IX complex need to be re-investigated in future studies. In our present studies, we investigated primarily the regulation of GPIbα-mediated signaling and showed that both iloprost (cAMP pathway) and riociguat (cGMP pathway) dose-dependently inhibited EB-induced aggregation of washed human platelets, with maximal inhibition at 5 nM iloprost and 20 μM riociguat, respectively. These clinically-used drugs and our conditions have been extensively used, and characterized to achieve strong, but selective activation of PKA (iloprost) and PKG (riociguat) in human platelets, also monitored by established substrates [8, 52–54]. Using different concentrations of iloprost (1 nM, 2 nM) and riociguat (10 μM, 20 μM), which inhibited significantly EB-induced aggregation, they did not inhibit, but instead enhanced/prolonged EB-induced Syk activation observed as Syk Y352 and Y525/526 phosphorylation (hyperphosphorylation). Enhanced phosphorylation of the direct Syk substrates PLCγ2 (Y759) and LAT (Y191) was also detected. In contrast, EB-induced Akt S473 phosphorylation was abolished. These results show that the PKA/PKG pathways do not prevent EB-induced Syk activation (Y352/ Y525/526) and Syk activity (PLCγ2/LAT), whereas further downstream effects (Akt phosphorylation, platelet aggregation) are strongly inhibited. The observation that cAMP and cGMP abolish GPIbα-mediated aggregation but not Syk activation under these conditions suggests that the inhibitory PKA/PKG effects occur downstream of Syk activation, not at the level of Syk activation/Syk activity.

Syk-mediated PLCγ2 activation and InsP3 and 1,2-diacylglycerol (DAG) generation are essential components of the GPVI-pathway in human and murine platelet, and most GPVI/ITAM-effects are thought be mediated by Syk [10, 13, 18], perhaps with some special exceptions such as generation of reactive oxygen species [64]. In contrast, specific GPIbα-stimulation of the Syk/PLCγ2 system with InsP3/DAG generation/intracellular Ca^2+^-release and its functional roles remained unclear until now [20, 30, 35, 65]. Our data conclusively show that EB, via GPIbα−/Syk activation, induced a marked increase of InsP1 as marker of InsP3 generation and Ca^2+^-release in human platelets, which was completely GPIbα-dependent (response abolished by EM), Syk-dependent (abolished by PRT-318) and integrin α_IIb_β_3_-independent (unaffected by tirofiban). However, iloprost (cAMP) and riociguat (cGMP) pre-treatment only partially inhibited EB-stimulated InsP1 increase and Ca^2+^-release. These only partial inhibitory effects differ from the known strong PKA/PKG-mediated inhibition of the InsP3/Ca^2+^ response by platelet GPCR (ADP, thrombin, TxA_2_) activation, which occurs at several sites including GPCR signaling, PLCβ, and the InsP3 receptor or its associated protein IRAG [8, 9, 61, 66, 67]. ADP−/ thrombin- / TxA_2_-stimulated Ca^2+^-release from intracellular stores in human platelets was essentially abolished by the PKA and PKG pathway [66, 67]. Our present data therefore suggest that cAMP/cGMP inhibit EB/GPIbα-platelet activation partially at the level of the InsP3/Ca^2+^ response but perhaps stronger at sites downstream of InsP3 production and Ca^2+^ elevation. Interestingly, earlier studies using PLCγ2-deficient murine platelets and vWF/botrocetin suggested that PLCγ2 is not required [35] or only moderately involved in GPIb-V-IX signaling [65] indicating that additional and/or compensating mechanisms exist, perhaps activation of other phospholipases. In our present studies, EB-induced aggregation, PLCγ2 Y759 phosphorylation, Akt S473 phosphorylation as well as InsP1 accumulation/InsP3 generation and Ca^2+^-release were found to be dependent on the secondary mediators ADP and TxA_2,_ different from the initial Syk phosphorylation/activation. It is very likely that important functional responses, such as Ca^2+^-release from intracellular stores, are heterogeneous and controlled by several pathways and proteins. A possible hotspot for the integration of various in-going Syk signals and out-going responses is the membrane protein LAT, an important Syk substrate and component of ITAM/Syk signaling in immune cells and also in platelets [10, 68].

Interestingly, we observed similar effects of cAMP/PKA and cGMP/PKG pathway stimulation on GPVI-mediated Syk phosphorylation. Whereas convulxin-stimulated platelet aggregation was completely inhibited by iloprost (cAMP) and riociguat (cGMP), convulxin-induced Syk phosphorylation (Y352 and Y525/526) was not inhibited, but instead clearly enhanced/prolonged (hyperphosphorylation) by iloprost or riociguat pretreatment. Convulxin-stimulated PLCγ2 Y759 phosphorylation was also enhanced by the cAMP/cGMP pathways, whereas Akt S473 phosphorylation was inhibited. In line with this observation, stimulation of CLEC-2 (a hemi-ITAM containing special adhesion receptor) on activated mouse platelets was shown to be only weakly inhibited by the cAMP, and not by the cGMP pathway [69]. Our cvx/GPVI signaling data agree with the GPVI-signaling data of a recent published study, which reported that the cAMP/PKA pathway inhibited distal but not proximal collagen/GPVI-mediated signaling events in human platelets [70].

Presently, the mechanism(s) for the PKA/PKG-mediated increase of Syk Y352 and Y525/526 phosphorylation (hyperphosphorylation) and kinase activity is a topic of further investigation in our laboratory. Interestingly, a possibly related “Syk Y-hyperphosphorylation” has been observed in response to PKC inhibitors [71]. Recently, a large spectrum of additional Syk serine and tyrosine phosphorylation sites, interacting proteins and multisite ubiquitylation has been described [46, 72]. Clearly, additional regulatory mechanisms exist, which may control phosphorylation/dephosphorylation and membrane interactions of Syk, and which need to be addressed in future studies.

**Fig. 9:**
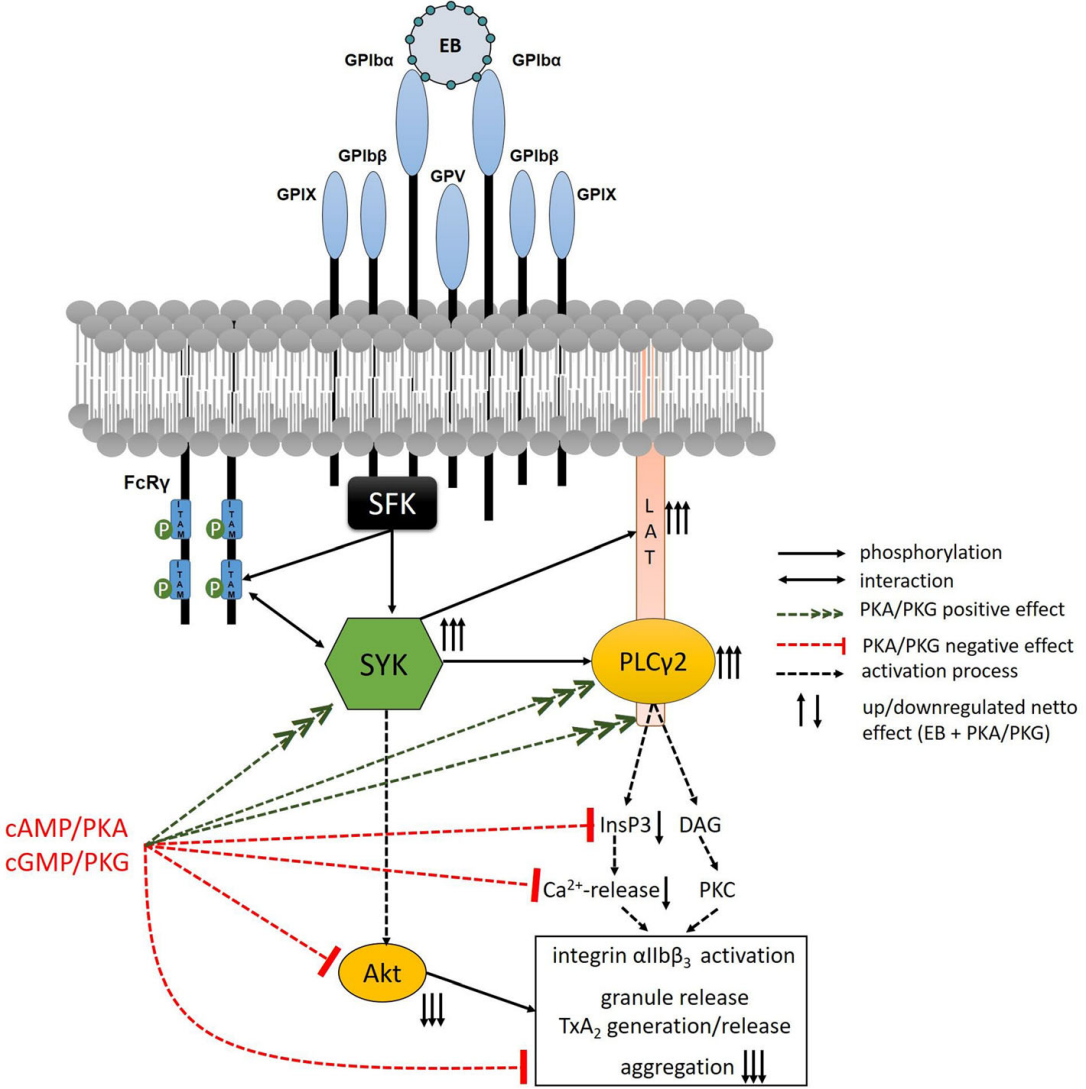
cAMP/PKA and cGMP/PKG pathways cause dichotomous regulation of GPIbα-mediated Syk stimulation and activation of human platelets. Selective binding of the snake venom toxin echicetin as multimeric complex (echicetin beads, EB) to the extracellular domain of GPIbα leads to GPIbα crosslinking and initiates a signaling cascade starting from activation of src family kinases (SFK). This GPIbα activation results in tyrosine phosphorylation of ITAM-containing FcRγ-chains and SFK-dependent phosphorylation and recruitment of the spleen tyrosine kinase (Syk), via its SH2 domains to tyrosine phosphorylated ITAMs produce full Syk activation. Syk-dependent phosphorylation and activation of the adaptor protein (LAT), phospholipase Cγ2 and others lead to increased levels of InsP3 (IP3; measured here by its metabolite InsP1) and DAG, which are responsible for Ca^2+^-release and PKC activation (dotted black arrows). Additionally, Syk mediates indirectly the phosphorylation of Akt, one of Syk downstream effectors (dotted black arrow). Altogether, this leads to integrin activation, granule release and TxA2 synthesis and subsequent platelet aggregation. The major platelet inhibitory pathways represented by cAMP/PKA and cGMP/PKG strongly enhance EB-induced Syk phosphorylation/activation (dotted green arrows) and enhance Syk-mediated tyrosine phosphorylation of LAT and PLCγ2 whereas InsP3 increase and Ca^2+^-release are partially inhibited, Akt phosphorylation is strongly inhibited (dotted red bars). The net effect of this crosstalk between platelet activation by EB and inhibition by cAMP/PKA and cGMP/PKG is marked with up or down black arrows (arrow number reflects the intensity of the effect). Syk and its direct substrates PLCγ2 and LAT are strongly activated (three arrows direction up), InsP3 and its subsequent Ca^2+^-release are partially inhibited (one arrow down). The phosphorylation of Akt is strongly inhibited by PKA and PKG-elevating agents, similar to the overall aggregation (three arrows direction down)

In contrast to Syk tyrosine phosphorylation, further downstream effects such as PI3K activation/Akt and aggregation in response to EB or convulxin were strongly inhibited by the PKA/PKG pathways or by blocking the secondary mediators ADP/TXA_2_. Both PKA/PKG pathways block platelet activation by ADP, TxA_2_, thrombin [8, 9, 67], which explains the inhibitory PKA/PKG effects on those components of GPIbα−/GPVI-induced platelet activation, which are dependent on secondary mediators. Previously it was suggested that several adhesion responses such as PI3K-dependent Akt activation are regulated by ADP-dependent and ADP-independent mechanisms [10, 73]. PKA and PKG are expected to inhibit ADP-dependent, but not ADP-independent PI3K/Akt activation. Finally, it has been increasingly recognized that crucial platelet functions are controlled and inhibited at multiple checkpoints including several PKA and PKG checkpoints [8, 9, 61]. A model of our current understanding of the effect of cAMP/PKA and cGMP/PKG on the GPIbα-regulated Syk network in human platelets is shown in Fig. 9.

## Conclusion

Our data establish that selective activation of GPIbα in human platelets by using echicetin-beads results in SFK-dependent Syk activation, subsequent Syk-mediated signaling, and ultimately integrin-dependent aggregation. In contrast to others [37] we obtained evidence that Syk plays an important role in EB- as well in ristocetin/vWF-mediated GPIbα-signaling of human platelets.

Syk pathway components display a variable dependency on secondary mediators (ADP, TxA2) and variable regulation by cAMP/cGMP inhibitory pathways, revealing two distinct directions of GPIbα−/Syk signaling. The initial GPIbα-caused Syk phosphorylation, activation and direct substrate phosphorylation is independent of the integrin αIIbβ3 and secondary mediators. Then, two distinct platelet inhibitory mediators, cAMP/PKA and cGMP/PKG, completely block one part of GPIbα−/GPVI-stimulated Syk signaling responses (aggregation, PI3K pathway), but increase the initial receptor-induced Syk activation and phosphorylation of two important direct Syk substrates, PLCγ2 and LAT. The observation that cAMP and cGMP abolish GPIbα-mediated aggregation but not Syk activation under our conditions suggests that the inhibitory PKA/PKG effects occur downstream of Syk activation, not at the level of Syk activation/Syk activity. Our results indicate the presence of distinct Syk signaling/effector systems, which differ in their interaction with other signaling pathways. The physiological and pathophysiological significance of these different Syk effector systems needs further investigation. This is clearly warranted in the light of the functional importance of Syk in platelets and immune cells, and in the light of Syk and related tyrosine protein kinases as important pharmacological target.

## Additional files

Additional file 1: **Figure S1.** Echicetin purification and qualitative validation. Echicetin was isolated from the snake venom of *Echis carinatus sochureki* by affinity chromatography followed by anion exchange chromatography using DEAE column. **a** Elution was performed with a gradient of NaCl, from 0 to 1 M at a flow rate of 1 ml/min. Two main peaks were separately eluted (P1 and P2) at around 62.5 and 167 mM, respectively. **b** Samples from P1 and P2 were analyzed by silver staining under non-reducing (NR) and reducing conditions (R) in a 15% SDS-gel. Bands detected under reducing conditions were cut from the gel, digested using trypsin, and analyzed by MS-analysis. The upper and lower bands of peak 1 were identified under the Uniprot IDs: P81017 (echicetin α-subunit) and P81996 (echicetin β-subunit), respectively. However, the upper band of peak 2 was detected as a sequence not related to *Echis carinatus sochureki* species, and the lower band was identified under the Uniprot ID: P81996 (echicetin β-subunit). For all experiments, echicetin from peak 1 was used. **c** Representative aggregation curves of human washed platelets (WP), which were stimulated under stirring conditions with EB or BSA-coated beads (as negative control). WP were pre-incubated with echicetin (EM) (25 μg/ml; 3 min) or with tirofiban (1.25 μg/ml; 1 min) prior to stimulation with EB. **d** Corresponding quantitative data of platelet aggregation expressed as maximum percentage of light transmission. Results are shown as means ± S.D. of 3 independent experiments with platelets from 3 healthy donors (****p < 0.0001). (PPTX 520 kb)

Additional file 2: **Figure S2.** Echicetin monomer does not affect aggregation induced by GPVI or by G-protein coupled receptors. Washed platelets were pre-incubated with 25 μg/ml echicetin monomer for 3 min before stimulating with **a** collagen (3 μg/ml), **c** ADP (1.75 μM), **e** TxA2 (60 nM), **g** TRAP6 (5 μM) and **i**-**k** thrombin (0.1 or 0.03 U/ml). Quantitative data are presented as means ± SD for **b** collagen, **d** ADP, **f** TxA2, **h** TRAP-6, **j**,**l** thrombin. Data are presented from 3 different experiments with platelets from 3 different healthy donors; ns: not significant. (PPTX 174 kb)

Additional file 3: **Figure S3.** EB-induced platelet aggregation is dependent on src family kinases (SFKs). Washed human platelets (WP) were pre-incubated for 5 min with the SFK inhibitor, PP2 (10 μM) or with its inactive analogue, PP3 (10 μM) prior to stimulation with EB. **a** Representative curves of the effect of PP2 and PP3 on platelet aggregation and **b** the corresponding quantification are shown as means ± S.D. Data are from at least 3 independent experiments with platelets from at least 3 healthy donors; ns: not significant, ****p < 0.0001. (PPTX 70 kb)

Additional file 4: **Figure S4.** Syk inhibitors diminish EB-induced general tyrosine phosphorylation and vWF/ristocetin-induced platelet aggregation and phosphorylation of Syk 525/526, LAT Y191 and Akt S473. **a** Washed human platelets (WP) were pre-incubated with 2 different Syk inhibitors, OXSI-2 (2 μM) and PRT-318 (1 μM) for 5 min prior to stimulation with EB. Aggregation was stopped after 1, 2 or 5 min using Laemmli buffer. General tyrosine phosphorylation was analyzed by western blot using a pan-phosphotyrosine antibody. **b** WP were pre-incubated for 5 min with vehicle control or PRT-318 (1 μM) in the presence or absence of 1.25 μg/ml tirofiban prior to stimulation with 2.5 μg/ml human vWF plus 0.5 mg/ml ristocetin. Representative curves show the effect of PRT-318 on vWF/R-induced platelet aggregation. Incubation of WP with tirofiban was used to dissect between vWF-mediated platelet agglutination and integrin α_IIb_β_3_-dependent aggregation. **c** The corresponding quantification demonstrates the effect of PRT-318 on the residual platelet aggregation response (overall maximum light transmission in the absence of tirofiban minus maximum light transmission in the presence of tirofiban) induced by vWF/risotcetin. Results are calculated from 4 different experiments with platelets from 4 healthy volunteers and data are presented as means ± S.D. **p < 0.01, ***p < 0.001, ****p < 0.0001. **d**, **e** Platelet aggregation was stopped after 2 min by addition of Laemmli buffer to analyze Syk Y525/526, Y352 and LAT Y191 and aggregation was stopped after 5 min for the analysis of Akt S473 by immunoblotting. Western blots are representative for at least 3 independent experiments from at least 3 healthy volunteers. (PPTX 380 kb)

Additional file 5: **Figure S5.** Iloprost and riociguat inhibit EB-induced platelet aggregation in a dose-dependent manner and induce stable VASP S157 and S239 phosphorylation. Representative curves of the effect of increased concentrations of **a** iloprost and **b** riociguat on EB-induced platelet aggregation. **c** Washed human platelets (WP) were pre-incubated for 3 and 2 min with 2 nM iloprost and 20 μM riociguat, respectively prior to stimulation with EB. VASP phosphorylation at S239 (the PKG preferred, but also PKA site) was analyzed by immunoblotting. VASP S157 phosphorylation at S157 (the PKA preferred, but also PKG site) is visible here by the well-established pS157-dependent shift from the 46 kDa to 50 kDa form of VASP in SDS-PAGE. The complete shift of the VASP to the 50 kDa form indicates near stoichiometric VASP S157 phosphorylation by iloprost-activated PKA. Quantification VASP S239 is represented as ratio compared to the loading control β-actin. Data are presented of 3 different experiments with platelets from 3 healthy volunteers as means ± S.D. ****p < 0.0001. (PPTX 292 kb)

Additional file 6: **Figure S6.** Syk and PLCγ2 tyrosine phosphorylation is also increased and prolonged at 1 nM iloprost. Washed human platelets (WP) were pre-incubated 3 min at 37 °C in the presence or absence of iloprost (1 nM) prior to stimulation with EB. Phosphorylation of **a** Syk Y525/526 and Y352, **d** PLCγ2 Y759 and Akt S473 was analyzed by immunoblotting in a time dependent manner. **b**, **c**, **e**, **f** Quantification of the phosphorylated proteins are represented as ratio compared to the corresponding total protein. Data are shown of 3 different experiments with platelets from 3 healthy volunteers as means ± S.D. ns: not significant, *p < 0.05, **p < 0.01, ****p < 0.0001. (PPTX 1770 kb)

Additional file 7: **Figure S7.** Syk and PLCγ2 tyrosine phosphorylation is also increased and prolonged at 10 μM riociguat. Washed human platelets (WP) were pre-incubated for 5 min at 37 °C with vehicle control (DMSO) and 10 μM riociguat, respectively prior to stimulation with EB. Phosphorylation of **a** Syk Y525/526 and Y352, **d** PLCγ2 Y759 and Akt S473 were analyzed by immunoblotting in a time dependent manner. **b**, **c**, **e**, **f** Quantification of the phosphorylated proteins are represented as ratio compared to the corresponding total protein. Data are shown of 3 different experiments with platelets from 3 healthy volunteers as means ± S.D. ns: not significant, *p < 0.05, **p < 0.01, ***p < 0.001, ****p < 0.0001. (Samples were loaded on the same gel; a black line was used to indicate that a group of samples not related to this data set was not shown). (PPTX 358 kb)

Additional file 8: **Figure S8.** Iloprost and riociguat inhibit convulxin-induced platelet aggregation but not Syk activation. Washed human platelets (WP) were pre-incubated with iloprost (2 nM, 3 min) or riociguat (20 μM, 2 min) prior to stimulation with 50 ng/ml convulxin. **a** Representative aggregation curves and the corresponding quantitative data are shown in **b**. **c** Aggregation was stopped after 1, 2 or 5 min using Laemmli buffer. Syk Y525/526 and Y352 were analyzed by western blot. **d**, **e** Quantification of the phosphorylated proteins is presented as ratio compared to the total Syk protein. Results are representative of at least 3 different experiments with platelets from at least 3 healthy volunteers, data are presented as means ± S.D. ns: not significant, ****p < 0.0001. (PPTX 181 kb)

## Data Availability

Datasets and non-commercial materials can be obtained from the corresponding author on reasonable request.

## Abbreviations

ADP: Adenosine diphosphate
Akt: Protein kinase B
cAMP: cyclic Adenosine Monophosphate
cGMP: cyclic Guanosine Monophosphate
CLEC-2: C-type lectin-2
EB: Echicetin Beads
EM: Echicetin Monomers
GPCRs: G protein-coupled receptors
GPIbα: Glycoprotein Ibα
InsP1/3: Inositol monophosphate/ triphosphate
ITAM: Immunoreceptor tyrosine-based activation motif
kDa: kilo Dalton
LAT: Linker of Activated T cells
NO: Nitric Oxide
PGI_2_: Prostaglandin I_2_
PKA: Protein Kinase A
PKG: Protein Kinase G
PLCγ: Phospholipase Cγ
SFK: Src- Family Kinase
Src: Sarcoma
Syk: Spleen tyrosine kinase
TxA_2_: Thromboxane A_2_
vWF: von Willebrand Factor
