# Supplementary figures and images for: cAMP- and cGMP-elevating agents inhibit GPIbα-mediated aggregation but not GPIbα-stimulated Syk activation in human platelets
Source: Cell Commun Signal. 2019 Sep 13;17:122. doi: 10.1186/s12964-019-0428-1 (PMC6743169; doi:10.1186/s12964-019-0428-1)

## Slide 1
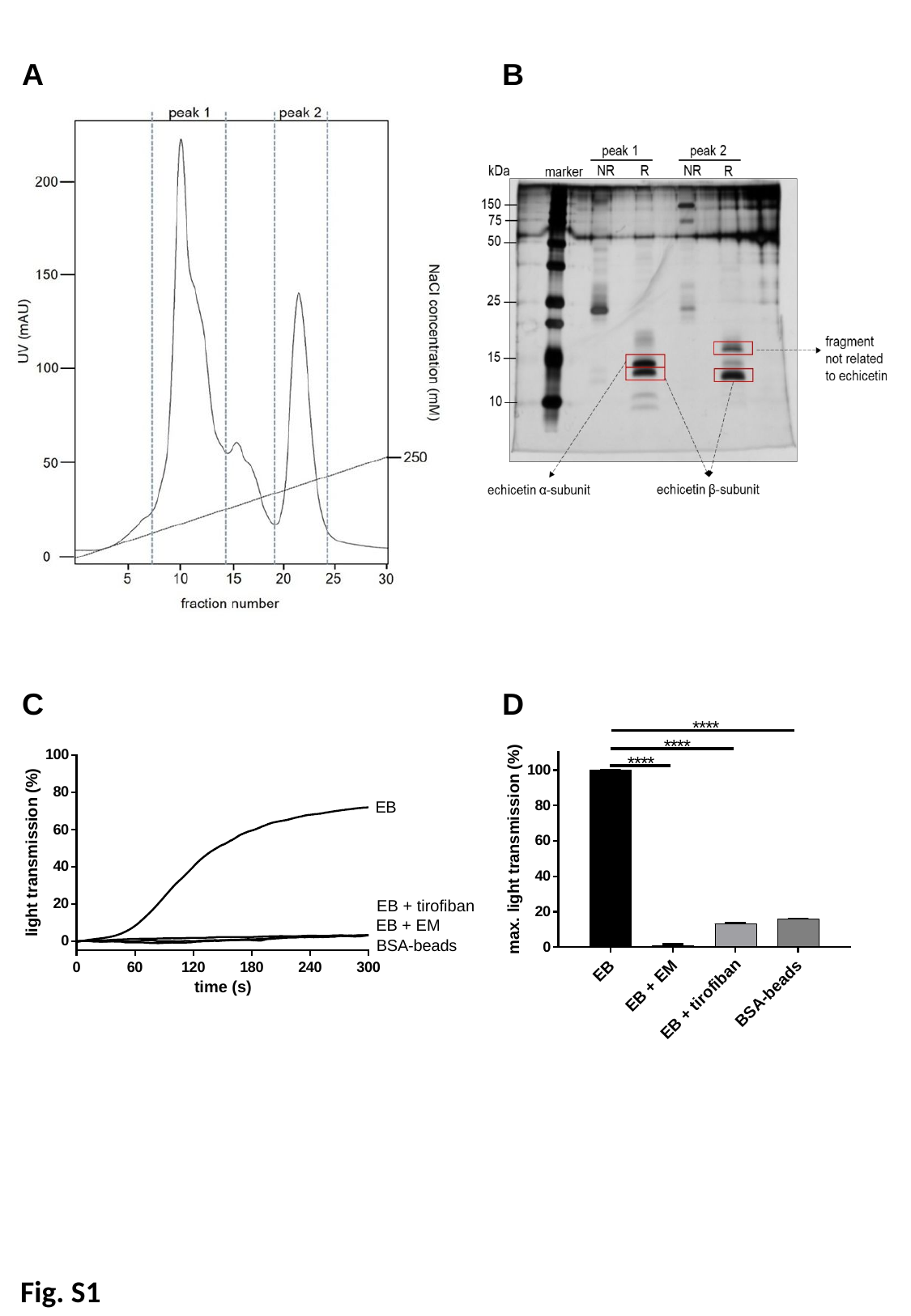

A
B
C
D
Fig. S1

Supplement: Supplementary file 1 — Figure S1. Echicetin purification and qualitative validation. Echicetin was isolated from the snake venom of Echis carinatus sochureki by affinity chromatography followed by anion exchange chromatography using DEAE column. a Elution was performed with a gradient of NaCl, from 0 to 1 M at a flow rate of 1 ml/min. Two main peaks were separately eluted (P1 and P2) at around 62.5 and 167 mM, respectively. b Samples from P1 and P2 were analyzed by silver staining under non-reducing (NR) and reducing conditions (R) in a 15% SDS-gel. Bands detected under reducing conditions were cut from the gel, digested using trypsin, and analyzed by MS-analysis. The upper and lower bands of peak 1 were identified under the Uniprot IDs: P81017 (echicetin α-subunit) and P81996 (echicetin β-subunit), respectively. However, the upper band of peak 2 was detected as a sequence not related to Echis carinatus sochureki species, and the lower band was identified under the Uniprot ID: P81996 (echicetin β-subunit). For all experiments, echicetin from peak 1 was used. c Representative aggregation curves of human washed platelets (WP), which were stimulated under stirring conditions with EB or BSA-coated beads (as negative control). WP were pre-incubated with echicetin (EM) (25 μg/ml; 3 min) or with tirofiban (1.25 μg/ml; 1 min) prior to stimulation with EB. d Corresponding quantitative data of platelet aggregation expressed as maximum percentage of light transmission. Results are shown as means ± S.D. of 3 independent experiments with platelets from 3 healthy donors (****p < 0.0001). (PPTX 520 kb) [file 12964_2019_428_MOESM1_ESM.pptx]

## Slide 1
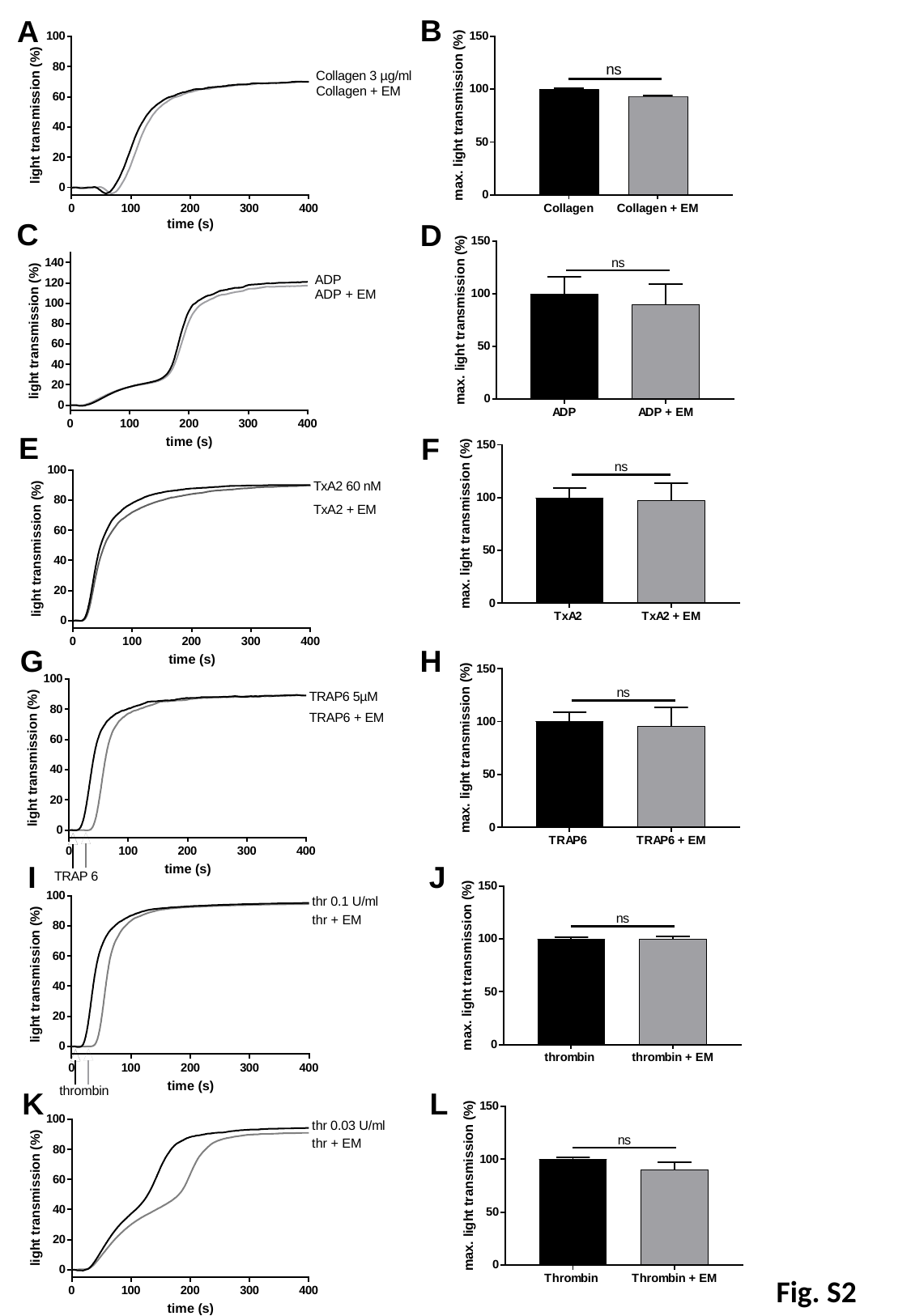

B
A
C
D
E
F
G
H
I
J
K
L
Fig. S2

Supplement: Supplementary file 2 — Figure S2. Echicetin monomer does not affect aggregation induced by GPVI or by G-protein coupled receptors. Washed platelets were pre-incubated with 25 μg/ml echicetin monomer for 3 min before stimulating with a collagen (3 μg/ml), c ADP (1.75 μM), e TxA2 (60 nM), g TRAP6 (5 μM) and i-k thrombin (0.1 or 0.03 U/ml). Quantitative data are presented as means ± SD for b collagen, d ADP, f TxA2, h TRAP-6, j,l thrombin. Data are presented from 3 different experiments with platelets from 3 different healthy donors; ns: not significant. (PPTX 174 kb) [file 12964_2019_428_MOESM2_ESM.pptx]

## Slide 1
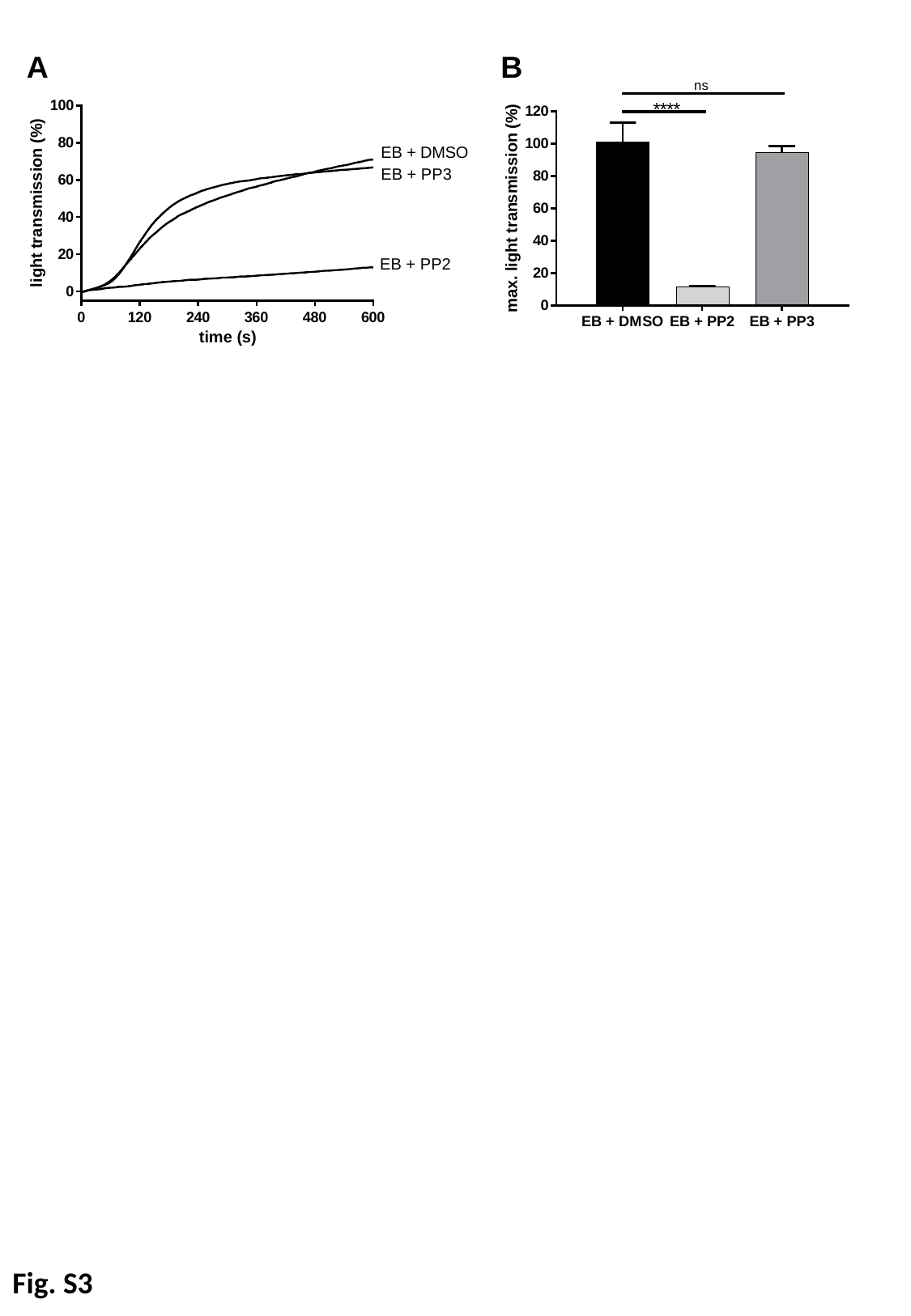

A
B
Fig. S3

Supplement: Supplementary file 3 — Figure S3. EB-induced platelet aggregation is dependent on src family kinases (SFKs). Washed human platelets (WP) were pre-incubated for 5 min with the SFK inhibitor, PP2 (10 μM) or with its inactive analogue, PP3 (10 μM) prior to stimulation with EB. a Representative curves of the effect of PP2 and PP3 on platelet aggregation and b the corresponding quantification are shown as means ± S.D. Data are from at least 3 independent experiments with platelets from at least 3 healthy donors; ns: not significant, ****p < 0.0001. (PPTX 70 kb) [file 12964_2019_428_MOESM3_ESM.pptx]

## Slide 1
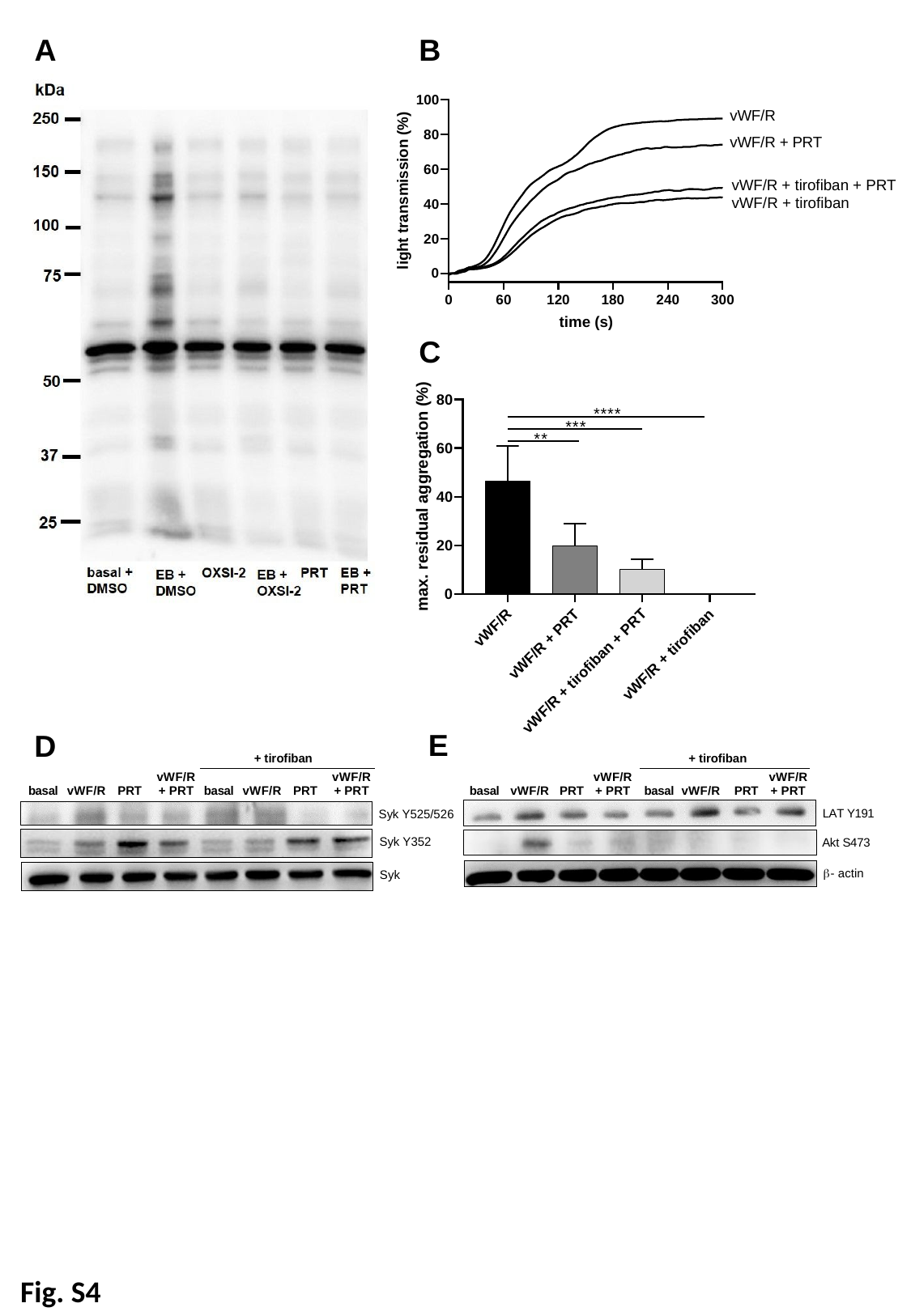

A
B
C
E
D
Fig. S4

Supplement: Supplementary file 4 — Figure S4. Syk inhibitors diminish EB-induced general tyrosine phosphorylation and vWF/ristocetin-induced platelet aggregation and phosphorylation of Syk 525/526, LAT Y191 and Akt S473. a Washed human platelets (WP) were pre-incubated with 2 different Syk inhibitors, OXSI-2 (2 μM) and PRT-318 (1 μM) for 5 min prior to stimulation with EB. Aggregation was stopped after 1, 2 or 5 min using Laemmli buffer. General tyrosine phosphorylation was analyzed by western blot using a pan-phosphotyrosine antibody. b WP were pre-incubated for 5 min with vehicle control or PRT-318 (1 μM) in the presence or absence of 1.25 μg/ml tirofiban prior to stimulation with 2.5 μg/ml human vWF plus 0.5 mg/ml ristocetin. Representative curves show the effect of PRT-318 on vWF/R-induced platelet aggregation. Incubation of WP with tirofiban was used to dissect between vWF-mediated platelet agglutination and integrin αIIbβ3-dependent aggregation. c The corresponding quantification demonstrates the effect of PRT-318 on the residual platelet aggregation response (overall maximum light transmission in the absence of tirofiban minus maximum light transmission in the presence of tirofiban) induced by vWF/risotcetin. Results are calculated from 4 different experiments with platelets from 4 healthy volunteers and data are presented as means ± S.D. **p < 0.01, ***p < 0.001, ****p < 0.0001. d, e Platelet aggregation was stopped after 2 min by addition of Laemmli buffer to analyze Syk Y525/526, Y352 and LAT Y191 and aggregation was stopped after 5 min for the analysis of Akt S473 by immunoblotting. Western blots are representative for at least 3 independent experiments from at least 3 healthy volunteers. (PPTX 380 kb) [file 12964_2019_428_MOESM4_ESM.pptx]

## Slide 1
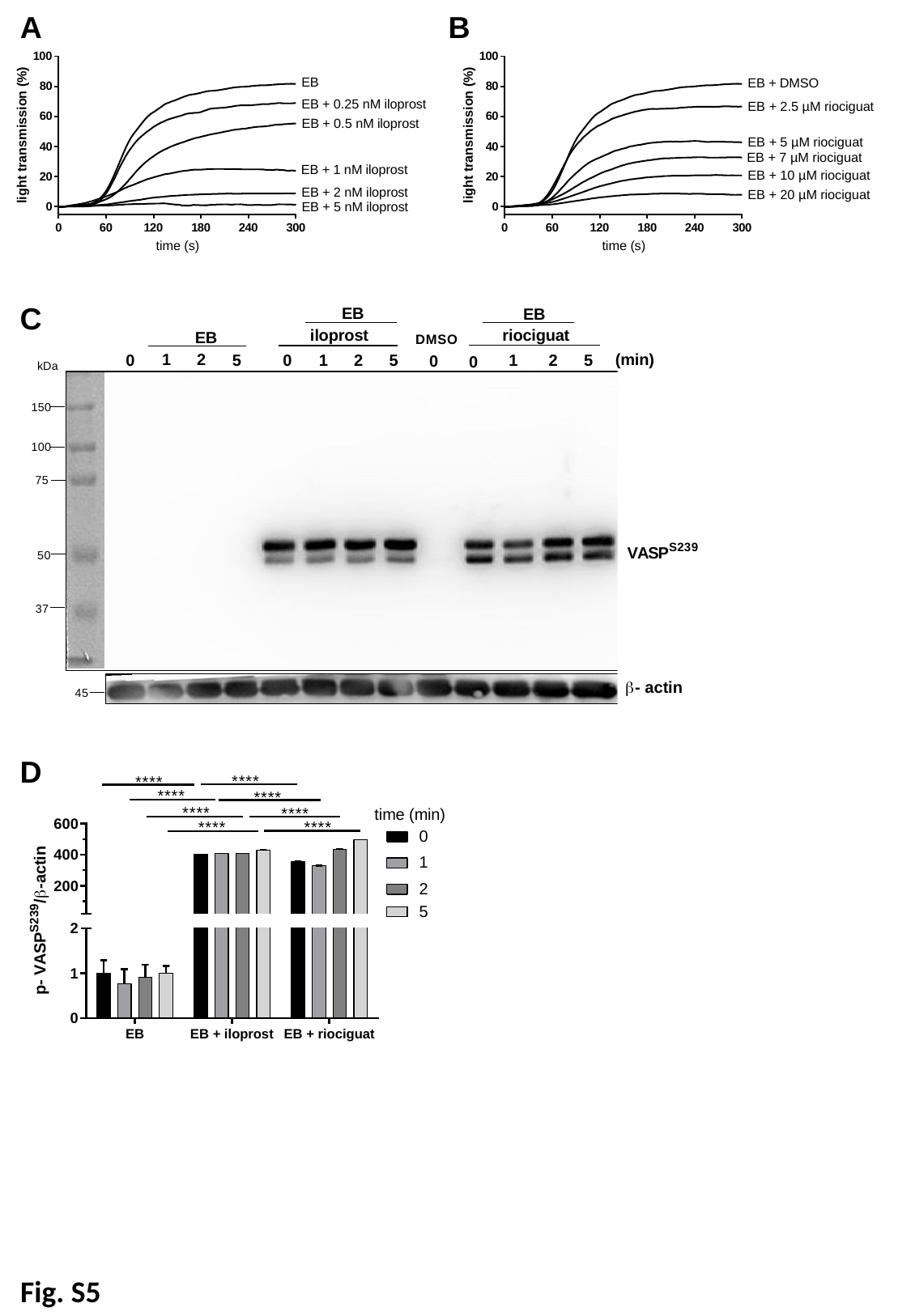

A
B
C
D
Fig. S5

Supplement: Supplementary file 5 — Figure S5. Iloprost and riociguat inhibit EB-induced platelet aggregation in a dose-dependent manner and induce stable VASP S157 and S239 phosphorylation. Representative curves of the effect of increased concentrations of a iloprost and b riociguat on EB-induced platelet aggregation. c Washed human platelets (WP) were pre-incubated for 3 and 2 min with 2 nM iloprost and 20 μM riociguat, respectively prior to stimulation with EB. VASP phosphorylation at S239 (the PKG preferred, but also PKA site) was analyzed by immunoblotting. VASP S157 phosphorylation at S157 (the PKA preferred, but also PKG site) is visible here by the well-established pS157-dependent shift from the 46 kDa to 50 kDa form of VASP in SDS-PAGE. The complete shift of the VASP to the 50 kDa form indicates near stoichiometric VASP S157 phosphorylation by iloprost-activated PKA. Quantification VASP S239 is represented as ratio compared to the loading control β-actin. Data are presented of 3 different experiments with platelets from 3 healthy volunteers as means ± S.D. ****p < 0.0001. (PPTX 292 kb) [file 12964_2019_428_MOESM5_ESM.pptx]

## Slide 1
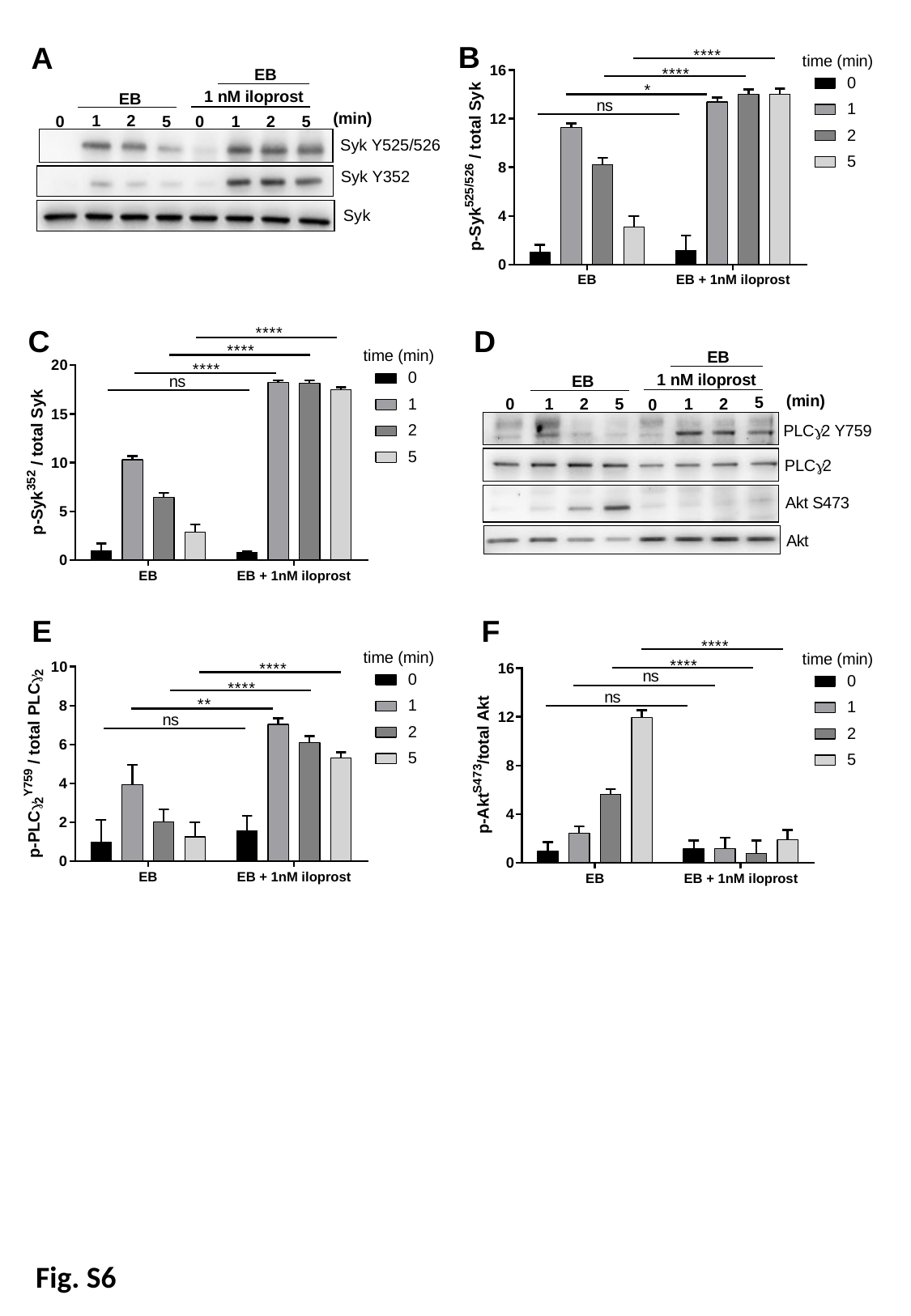

B
A
C
D
E
F
Fig. S6

Supplement: Supplementary file 6 — Figure S6. Syk and PLCγ2 tyrosine phosphorylation is also increased and prolonged at 1 nM iloprost. Washed human platelets (WP) were pre-incubated 3 min at 37 °C in the presence or absence of iloprost (1 nM) prior to stimulation with EB. Phosphorylation of a Syk Y525/526 and Y352, d PLCγ2 Y759 and Akt S473 was analyzed by immunoblotting in a time dependent manner. b, c, e, f Quantification of the phosphorylated proteins are represented as ratio compared to the corresponding total protein. Data are shown of 3 different experiments with platelets from 3 healthy volunteers as means ± S.D. ns: not significant, *p < 0.05, **p < 0.01, ****p < 0.0001. (PPTX 1770 kb) [file 12964_2019_428_MOESM6_ESM.pptx]

## Slide 1
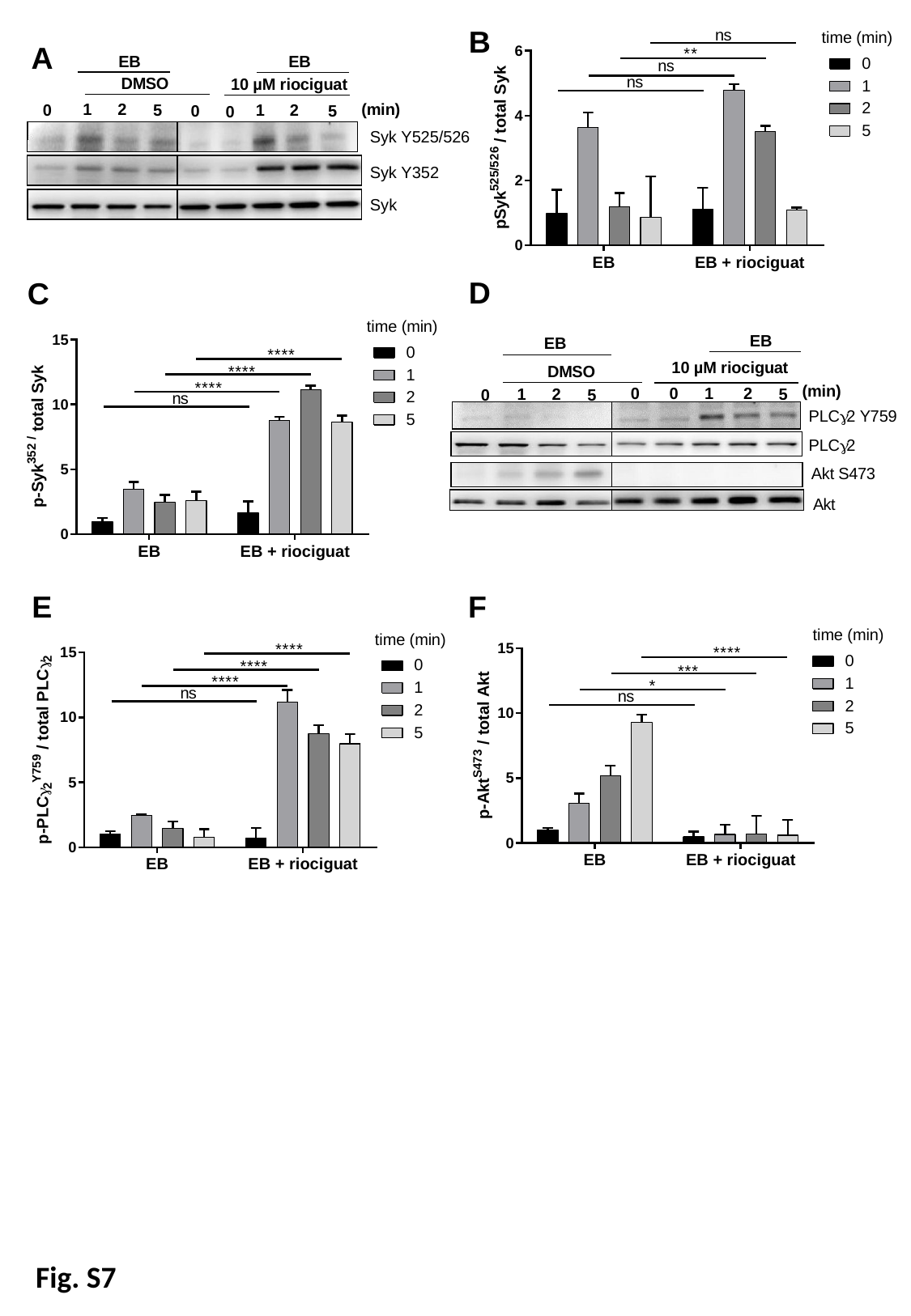

B
A
D
C
E
F
Fig. S7

Supplement: Supplementary file 7 — Figure S7. Syk and PLCγ2 tyrosine phosphorylation is also increased and prolonged at 10 μM riociguat. Washed human platelets (WP) were pre-incubated for 5 min at 37 °C with vehicle control (DMSO) and 10 μM riociguat, respectively prior to stimulation with EB. Phosphorylation of a Syk Y525/526 and Y352, d PLCγ2 Y759 and Akt S473 were analyzed by immunoblotting in a time dependent manner. b, c, e, f Quantification of the phosphorylated proteins are represented as ratio compared to the corresponding total protein. Data are shown of 3 different experiments with platelets from 3 healthy volunteers as means ± S.D. ns: not significant, *p < 0.05, **p < 0.01, ***p < 0.001, ****p < 0.0001. (Samples were loaded on the same gel; a black line was used to indicate that a group of samples not related to this data set was not shown). (PPTX 358 kb) [file 12964_2019_428_MOESM7_ESM.pptx]

## Slide 1
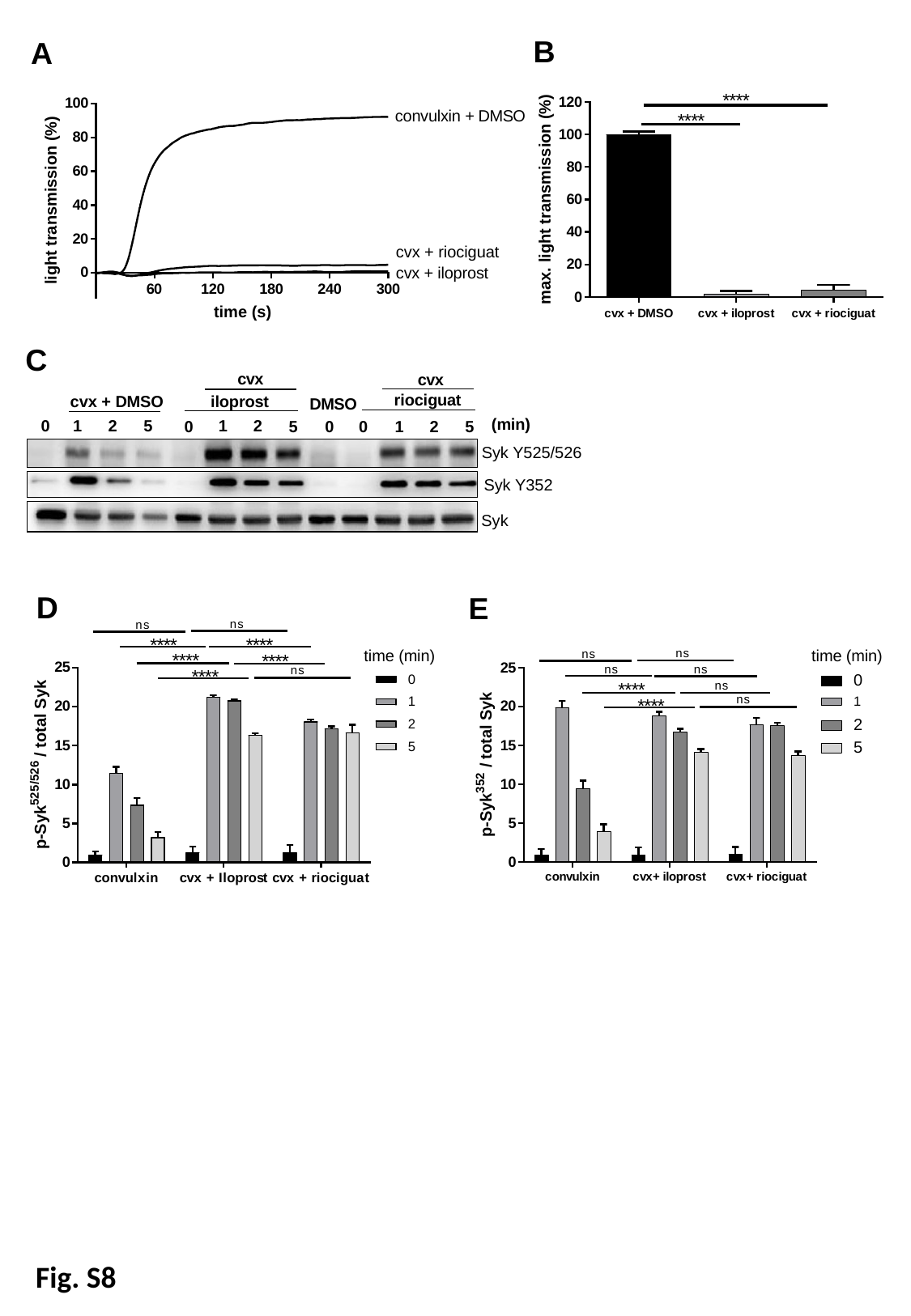

B
A
C
D
E
Fig. S8

Supplement: Supplementary file 8 — Figure S8. Iloprost and riociguat inhibit convulxin-induced platelet aggregation but not Syk activation. Washed human platelets (WP) were pre-incubated with iloprost (2 nM, 3 min) or riociguat (20 μM, 2 min) prior to stimulation with 50 ng/ml convulxin. a Representative aggregation curves and the corresponding quantitative data are shown in b. c Aggregation was stopped after 1, 2 or 5 min using Laemmli buffer. Syk Y525/526 and Y352 were analyzed by western blot. d, e Quantification of the phosphorylated proteins is presented as ratio compared to the total Syk protein. Results are representative of at least 3 different experiments with platelets from at least 3 healthy volunteers, data are presented as means ± S.D. ns: not significant, ****p < 0.0001. (PPTX 181 kb) [file 12964_2019_428_MOESM8_ESM.pptx]
